# mTORC1 Is Transiently Reactivated in Injured Nerves to Promote c-Jun Elevation and Schwann Cell Dedifferentiation

**DOI:** 10.1523/JNEUROSCI.3619-17.2018

**Published:** 2018-05-16

**Authors:** Camilla Norrmén, Gianluca Figlia, Patrick Pfistner, Jorge A. Pereira, Sven Bachofner, Ueli Suter

**Affiliations:** Institute of Molecular Health Sciences, Department of Biology, Swiss Federal Institute of Technology, ETH Zürich, Zürich CH-8093, Switzerland

**Keywords:** differentiation, injury, mTOR, myelination, nerve, Schwann cells

## Abstract

Schwann cells (SCs) are endowed with a remarkable plasticity. When peripheral nerves are injured, SCs dedifferentiate and acquire new functions to coordinate nerve repair as so-called repair SCs. Subsequently, SCs redifferentiate to remyelinate regenerated axons. Given the similarities between SC dedifferentiation/redifferentiation in injured nerves and in demyelinating neuropathies, elucidating the signals involved in SC plasticity after nerve injury has potentially wider implications. c-Jun has emerged as a key transcription factor regulating SC dedifferentiation and the acquisition of repair SC features. However, the upstream pathways that control c-Jun activity after nerve injury are largely unknown. We report that the mTORC1 pathway is transiently but robustly reactivated in dedifferentiating SCs. By inducible genetic deletion of the functionally crucial mTORC1-subunit Raptor in mouse SCs (including male and female animals), we found that mTORC1 reactivation is necessary for proper myelin clearance, SC dedifferentiation, and consequently remyelination, without major alterations in the inflammatory response. In the absence of mTORC1 signaling, c-Jun failed to be upregulated correctly. Accordingly, a c-Jun binding motif was found to be enriched in promoters of genes with reduced expression in injured mutants. Furthermore, using cultured SCs, we found that mTORC1 is involved in c-Jun regulation by promoting its translation, possibly via the eIF4F-subunit eIF4A. These results provide evidence that proper c-Jun elevation after nerve injury involves also mTORC1-dependent post-transcriptional regulation to ensure timely dedifferentiation of SCs.

**SIGNIFICANCE STATEMENT** A crucial evolutionary acquisition of vertebrates is the envelopment of axons in myelin sheaths produced by oligodendrocytes in the CNS and Schwann cells (SCs) in the PNS. When myelin is damaged, conduction of action potentials along axons slows down or is blocked, leading to debilitating diseases. Unlike oligodendrocytes, SCs have a high regenerative potential, granted by their remarkable plasticity. Thus, understanding the mechanisms underlying SC plasticity may uncover new therapeutic targets in nerve regeneration and demyelinating diseases. Our work reveals that reactivation of the mTORC1 pathway in SCs is essential for efficient SC dedifferentiation after nerve injury. Accordingly, modulating this signaling pathway might be of therapeutic relevance in peripheral nerve injury and other diseases.

## Introduction

Myelin diseases of the CNS and PNS are prevalent causes of morbidity and mortality worldwide. Together with therapies targeting primary causes of demyelination, many research efforts have focused on promoting the regenerative potential of myelinating glia and their progenitors (Plemel et al., 2017; Stangel et al., 2017; Franklin and ffrench-Constant, 2017). Unlike myelinating oligodendrocytes in the CNS, Schwann cells (SCs) in the PNS are highly plastic and account to a large extent for the high regenerative capabilities of nerves (Brosius Lutz and Barres, 2014). SCs dedifferentiate in response to injury, diseases, or genetic manipulations, and redifferentiate upon cessation of the initial trigger (Napoli et al., 2012; Boerboom et al., 2017; Gomez-Sanchez et al., 2017). A mechanistic understanding of SC plasticity may therefore reveal new targets to promote nerve regeneration, remyelination, or to prevent demyelination in neuropathies. Furthermore, such studies might inspire new strategies to improve the limited regenerative potential of the CNS.

Nerve injuries offer an experimental model to study SC dedifferentiation/redifferentiation (Stoll et al., 2002; Savastano et al., 2014). When nerves are injured, partially overlapping events ensue: (1) axons degenerate distal to the injury site; (2) SCs dedifferentiate; (3) inflammatory cells are recruited; (4) myelin breaks down; (5) axons regrow; and (6) SCs remyelinate the regenerated axons. The use of such models has revealed that SCs do not simply undergo dedifferentiation defined as reacquisition of the molecular phenotype of developing SCs. Instead, SCs also acquire new molecular features and functions to coordinate a sophisticated injury response, such as myelin autophagy and phagocytosis (Gomez-Sanchez et al., 2015; Jang et al., 2016; Brosius Lutz et al., 2017), secretion of cytokines to recruit inflammatory cells (Martini et al., 2008), and secretion of neurotrophic factors to support neuron survival and axonal regeneration (Meyer et al., 1992; Webber and Zochodne, 2010; Fontana et al., 2012). Hence, the term “repair SC” has been introduced to define the identity of SCs after injury (Jessen and Mirsky, 2016).

c-Jun plays a key role in reprogramming SCs to repair SCs. This transcription factor is strongly upregulated by dedifferentiating SCs. In its absence, SC dedifferentiation, myelin clearance, neuron survival, and axonal regeneration are impaired (Arthur-Farraj et al., 2012; Fontana et al., 2012). Intriguingly, c-Jun expression is elevated in human neuropathies and corresponding animal models, suggestive of a related role as in nerve injury (Hutton et al., 2011; Hantke et al., 2014). However, which signaling pathways regulate c-Jun expression in dedifferentiating SCs, and how, is unclear.

mTOR complex 1 (mTORC1) is the hub of a major pathway for cell growth. By phosphorylating several targets, mTORC1 promotes anabolism, including mRNA translation, synthesis of lipids, purines, and pyrimidines, while inhibiting autophagy (Ben-Sahra and Manning, 2017; Saxton and Sabatini, 2017). Additionally, alongside its direct growth-promoting functions, mTORC1 has a profound effect on the transcriptome (Düvel et al., 2010) and on cell fate decisions (Bulut-Karslioglu et al., 2016; Pollizzi et al., 2016; Verbist et al., 2016) by controlling transcription factors (Laughner et al., 2001; Roczniak-Ferguson et al., 2012; Tiebe et al., 2015; Park et al., 2017). In developing SCs, mTORC1 signaling is required for radial sorting of axons, lipid biosynthesis, and myelin growth (Sherman et al., 2012; Norrmén et al., 2014). Moreover, activity and function of mTORC1 change according to the SC differentiation status (Beirowski et al., 2017; Figlia et al., 2017, 2018). mTORC1 activity is high before onset of myelination and declines as myelination starts. Correspondingly, high mTORC1 activity inhibits differentiation to myelinating cells in not yet myelinating SCs through a mechanism involving S6K-dependent suppression of the master regulator of PNS myelination, Krox20. Conversely, mTORC1 supports myelin synthesis once SCs have started myelinating.

Here, we explored the function of mTORC1 in SCs after nerve injury. We propose a model in which transient but robust reactivation of mTORC1 is required for SC dedifferentiation and reprogramming to repair SCs through a process that involves increased translation of c-Jun.

## Materials and Methods

### 

#### 

##### Animal procedures.

Mice harboring floxed alleles of *Rptor* (Bentzinger et al., 2008; Polak et al., 2008), mice carrying a *Cre* transgene under control of the *Mpz* promoter (RRID:IMSR_JAX:017927), or a *CreERT2* transgene under control of the *Plp1* promoter have been described (Feltri et al., 1999; Jaegle et al., 2003; Leone et al., 2003). Mice harboring a Cre activity-reporting *tdTomato* transgene in the *Rosa26* locus (Madisen et al., 2010) were obtained from The Jackson Laboratory (B6.Cg-*Gt(Rosa)26Sor^tm9(CAG-tdTomato)Hze^*/J, catalog #007909, RRID:IMSR_JAX:007909). To generate inducible conditional deletion of Raptor, floxed mice were crossed with *Plp1^CreERT2^*-positive mice, and at 7–9 weeks of age 2 mg of tamoxifen (Sigma-Aldrich) in 10% ethanol/sunflower seed oil (Sigma-Aldrich) was injected intraperitoneally once a day on 5 consecutive days in both mutant and control animals. Experimental animals were backcrossed 6–9 times with C57B6/J. Cre-negative animals (floxed homozygous) were used as controls in the experiments. Wild-type mice were on a C57B6/J background. Mice of either sex were used in the experiments. Genotypes were determined through genomic PCR using the following primers: Cre forward 5′-accaggttcgttcactcatgg-3′, reverse 5′-aggctaagtgcctcttctaca-3′; Raptor forward 5′-atggtagcaggcacactcttcatg-3′, reverse 5′-gctaaacattcagtccctaatc-3′; Rosa26 wild-type forward 5′-aagggagctgcagtggagta-3′, reverse 5′-ccgaaaatctgtgggaagtc-3′; and Rosa26 tdTomato forward 5′-ggcattaaagcagcgtatcc-3′, reverse 5′-ctgttcctgtacggcatgg-3′. Mice were housed with a maximum number of 5 animals per cage, kept in a 12 h light-dark cycle, and fed standard chow *ad libitum*. All animal experiments were approved by the Veterinary Office of the Canton (Zürich, Switzerland).

##### Surgical procedures.

Mice were subjected to unilateral sciatic nerve crush injury 2 months after tamoxifen administration. After inducing anesthesia with isoflurane inhalation, the sciatic nerve was exposed through blunt dissection of the thigh muscles and compressed for 30 s. For analgesia, 0.1 mg/kg buprenorphine (Temgesic, Reckitt Benckiser) was injected intraperitoneally once before the surgery, and treatment was maintained for 2 d after injury. Morphological analysis and immunostaining of crushed nerves were performed on sections 3 mm distal to the injury site. For protein lysates and RNA extraction, the whole nerve distal to the injury site was used.

##### Morphological analysis and g-ratio measurements.

Immediately after dissection, sciatic nerves were fixed with 3% glutaraldehyde and 4% PFA in 0.1 m phosphate buffer. Sciatic nerves were further treated with 2% osmium tetroxide (EMS), dehydrated over a series of acetone gradients, and embedded in Spurrs resin (EMS). Semithin sections (650 nm) were stained with 1% toluidine blue and used for qualitative analysis. Ultrathin sections (65 nm) were imaged with an FEI Morgagni 268 TEM, and random 5 × 5 multiple image alignment fields were acquired for qualitative analysis. g-ratio measurements, quantification of remyelinated and total nerve fibers, and quantification of intact-appearing myelin profiles were performed on EM reconstructions of the entire sciatic nerve section, obtained from additional sections (99 nm) collected on ITO coverslips (Optics Balzers) and imaged with either a Carl Zeiss Gemini Leo 1530 FEG or Carl Zeiss Merlin FEG scanning electron microscopes attached to ATLAS modules (Carl Zeiss). To calculate the g-ratio (the ratio between axon diameter and fiber diameter), the axon diameter was derived from the axon area measured using Adobe Photoshop CS5 (Adobe, RRID:SCR_014199), whereas the fiber diameter was calculated adding to the axon diameter twice the average of the myelin thickness measured with Adobe Photoshop CS5 at two different locations of the myelin ring. At least 100 fibers per nerve were analyzed.

##### Antibodies and chemical inhibitors.

The following primary antibodies were used: phospho-S6K^T389^ (Cell Signaling Technology, catalog #9234S, RRID:AB_2269803, 1:1000), S6K (Cell Signaling Technology, catalog #9202 RRID:AB_331676, 1:1000), phospho-S6^S235/236^ (Cell Signaling Technology, catalog #4857S, RRID:AB_2181035, 1:1000 for Western blot, 1:200 for immunohistochemistry), S6 (Cell Signaling Technology, catalog #2317S, RRID:AB_10694551, 1:1000), c-Jun (Cell Signaling Technology, catalog #9165, RRID:AB_2130165, 1:1000), phospho-Akt^T308^ (Cell Signaling Technology, catalog #4056S, RRID:AB_331163, 1:1000), phospho-Akt^S473^ (Cell Signaling Technology, catalog #4051S, RRID:AB_331158, 1:1000), Akt (Cell Signaling Technology, catalog #9272S, RRID:AB_329827, 1:1000), P0 (Millipore, catalog #AB9352, RRID:AB_571090, 1:1000), cleaved caspase 3 (Cell Signaling Technology, catalog #9661S, RRID:AB_2341188, 1:500), phospho-Erk1/2^T202/Y204^ (Cell Signaling Technology, catalog #9106S, RRID:AB_331768, 1:1000), Erk1/2 (Cell Signaling Technology, catalog #9102S, RRID:AB_330744, 1:1000), α-tubulin (Sigma-Aldrich, catalog #T5168, RRID:AB_477579, 1:1000), β-actin (Sigma-Aldrich, catalog #A5316, RRID:AB_476743, 1:1000), GAPDH (Hytest, catalog #5G4–9B3, RRID:AB_1616725, 1:10,000), Sox10 (R&D Systems, catalog #AF2864, 1:100), Runx2 (Cell Signaling Technology, catalog #12556, 1:100), and CD68 (Serotec, catalog #MCA1957, RRID:AB_322219, 1:100). Antibodies against Oct6 (used 1:300) were a generous gift from Dr. Dies Meijer. HRP-, AP-, and fluorophore-conjugated secondary antibodies were purchased from Jackson ImmunoResearch Laboratories and used 1:200 for immunostainings or 1:10,000 for Western blots. The following chemical inhibitors were used: rapamycin (Sigma-Aldrich), Torin-1 (Tocris Bioscience), silvestrol (Sarawak Biodiversity Centre), and actinomycin D (Sigma-Aldrich).

##### Western blots.

Immediately after dissection, sciatic nerves were placed in ice-cold PBS, the epineurium was removed with fine forceps, and the nerves were snap-frozen in liquid nitrogen and stored at −80°C until further processing. To prepare lysates, nerves were ground on dry ice, mixed with PN2 lysis buffer (25 mm Tris-HCl, pH 7.4, 95 mm NaCl, 10 mm EDTA, 2% SDS, protease and phosphatase inhibitors; Roche Diagnostics), boiled, and spun for 15 min; 30–50 μg of proteins per sample was mixed 1:4 with sample buffer (200 mm Tris-HCl, pH 6.8, 40% glycerol, 8% SDS, 20% β-mercaptoethanol, 0.4% bromophenol blue), run on 4%–15% polyacrylamide gradient gels (Bio-Rad), and blotted onto PVDF membranes (Millipore). After blocking with 5% milk in TBS-T, membranes were incubated overnight with primary antibodies diluted in 5% BSA in TBS-T. Chemiluminescent signals were generated using HRP- or AP-conjugated secondary antibodies and ECL (GE Healthcare), ECL prime (GE Healthcare), or CDP-Star (Roche Diagnostics), and detected using Fusion FX7 (Vilber Lourmat). If required, after signal detection, membranes were stripped either with a low pH buffer (25 mm glycine-HCl, pH 2.2, 1% (w/v) SDS) for 20–30 min, or with a buffer containing β-mercaptoethanol (1.25 mm Tris-HCl, pH 6.8, 2% SDS, 100 mm β-mercaptoethanol) for 15 min at 55°C, and thereafter reprobed with antibodies. Quantification of band intensities was performed with ImageJ (version 1.50i). α-Tubulin, β-actin, or GAPDH was used as normalization controls. For cropped blots, the loading control bands are displayed immediately below the other bands that belong to the same membrane. Size markers refer to All Blue Precision Protein Standards (Bio-Rad). Protein size is expressed as apparent molecular weight in kDa.

##### Immunostaining.

Immediately after dissection, sciatic nerves were fixed for 1 h in 4% PFA, cryopreserved in 10% sucrose for 1 h, 20% sucrose overnight, and then embedded in OCT (TissueTek). The 10-μm-thick cryosections were cut, treated for 10 min with prechilled acetone (for Oct6 staining) or 4% PFA (for Sox10, Runx2, and CD68 staining), blocked with blocking buffer (1% BSA, 10% goat or donkey serum, 0.1% Triton X-100 in PBS) for 1 h, incubated overnight with primary antibodies, incubated for 1 h with fluorophore-conjugated secondary antibodies, and mounted with DAPI-containing Vectashield (Vector Laboratories). For cell proliferation experiments, mice were injected with 50 μg 5-ethynyl-2-deoxyuridine (EdU; Invitrogen) per gram of body weight and killed 1 h later. EdU staining was performed using the Click-iT EdU Alexa647 kit (Invitrogen) as per the manufacturer's instructions. Immunostainings were imaged using an epifluorescence microscope (Axioplan 2 or Axio Imager.M2, Carl Zeiss), equipped with a CCD camera (AxioCam MRm, Carl Zeiss, or sCMOS, pco.edge). One representative section per sample was imaged and analyzed. Oct6- and DAPI-positive nuclei were counted using the CellProfiler image analysis software (Broad Institute, RRID:SCR_007073), whereas CD68-, EdU-, Sox10-, and cleaved caspase 3 (cC3)-positive cells were counted manually. When necessary, image levels were adjusted using Photoshop CS6 (Adobe, RRID:SCR_014199).

##### RNA extraction and qRT-PCR analysis.

Immediately after dissection, sciatic nerves were placed in ice-cold PBS, the epineurium was removed with fine forceps, and the nerves were snap-frozen in liquid nitrogen and stored at −80°C until needed. Total RNA was extracted using Qiazol (QIAGEN) for mouse samples and cells as per the manufacturer's instructions; 50–200 ng of total RNA was reverse transcribed using Maxima First Strand cDNA Synthesis Kit (Thermo Fisher Scientific) as per the manufacturer's instructions. qPCRs were performed using FastStart Essential DNA Green Master (Roche Diagnostics) and Light Cycler 480 II (Roche Diagnostics). The sequences of the primers used and their specificities are as follows: c-Jun (mouse and rat) forward 5′-gccaagaactcggaccttctcacgtc-3′, reverse 5′-tgatgtgcccattgctggactggatg-3′; GDNF (mouse) forward 5′-cgctgaccagtgactccaat-3′, reverse 5′-gctgccgcttgtttatctgg-3′; Olig1 (mouse) forward 5′-accatgcggatctaggaggt-3′, reverse 5′-taacatccagctcggaaaccc-3′; Shh (mouse) forward 5′-aaagctgacccctttagccta-3′, reverse 5′-ttcggagtttcttgtgatcttcc-3′; Runx2 (mouse) forward 5′-ttcaacgatctgagatttgtggg-3′, reverse 5′-ggatgaggaatgcgcccta-3′; GAPDH (mouse and rat) forward 5′-ggtgaaggtcggtgtgaacggatttgg-3′, reverse 5′-ggtcaatgaaggggtcgttgatggcaac-3′; β-actin (mouse) forward 5′-ttctttgcagctccttcgtt-3′, reverse 5′-atggaggggaatacagccc-3′; and β-actin (rat) forward 5′-acaaccttcttgcagctcctc-3′, reverse 5′-gacccatacccaccatcacac-3′. Relative mRNA fold changes for each gene were obtained by using the 2^−ΔΔCt^ method after normalization to GAPDH or β-actin.

##### Preparation, culture, and use of primary SCs.

To prepare rat SCs (rSCs), sciatic nerves were dissected from P2 Sprague Dawley rat pups, the epineurium was removed, and nerves were digested with 1.25 mg/ml trypsin (Sigma-Aldrich, catalog #T9201) and 2 mg/ml collagenase (Sigma-Aldrich, catalog #C0130) for 1 h. After centrifugation, the cells were resuspended in D-medium (DMEM-Glutamax plus 10% FCS; Invitrogen) and plated on PLL-coated dishes. Contaminating fibroblasts were eliminated with 10 μm Ara-C treatment (Sigma-Aldrich) for 48 h, followed by complement-mediated removal of fibroblasts using an anti-Thy1.1 antibody (Bio-Rad, catalog #MCA04G, RRID:AB_322809, 1:50). After a 10 min incubation with the anti-Thy1.1 antibody, rabbit complement (Millipore) was added for 40 min. SCs were cultured at 37°C 5% CO_2_ in SC growth medium (DMEM-Glutamax; Invitrogen), 10% FCS (Invitrogen), 5 μg/ml bovine pituitary extract (Invitrogen), 2 μm forskolin (Sigma-Aldrich), tested for mycoplasma contamination, aliquoted, and stored in liquid nitrogen until needed. SCs were kept in growth medium for experiments. Cells were lysed with Qiazol (QIAGEN) for qRT-PCR, or PN2 lysis buffer mixed 1:4 with sample buffer for Western blot.

##### Nerve explants.

Distal segments (1 cm long) of sciatic nerves from wild-type mice at 4 months, or control and RaptorKO mice 2 months after tamoxifen administration were collected, transferred to DMEM-Glutamax (Invitrogen) supplemented with 5% FCS (Invitrogen), and incubated at 37°C 5% CO_2_ until ready for analysis. For Western blot analysis, the epineurium was removed immediately after dissection. To evaluate the rate of protein synthesis, a modified version of a puromycin-based assay was used. In this assay, puromycin is added cotranslationally to nascent polypeptides allowing to monitor the overall rate of protein synthesis (Schmidt et al., 2009). Briefly, nerve explants were incubated with puromycin (Sigma-Aldrich, 10 μg/ml) for 30 min immediately after dissection and removal of the epineurium (control), or after a 24 h treatment with DMSO or Torin 1 (250 nm). After incubation with puromycin, nerves were snap frozen, lysed, and subjected to Western blot analysis using an anti-puromycin antibody (Millipore, catalog #MABE343, RRID:AB_2566826, 1:1000) to detect newly synthesized proteins. Ponceaus S staining was used as loading control.

##### RNA sequencing.

The quantity and quality of isolated RNA were determined with a Qubit (1.0) Fluorometer (Invitrogen) and a Bioanalyzer 2100 (Agilent Technologies). The TruSeq Stranded mRNA Sample Prep Kit (Illumina) was used in the succeeding steps. Briefly, total RNA samples (100 ng) were ribosome depleted, reverse-transcribed into double-stranded cDNA with actinomycin added during first-strand synthesis. The cDNA samples were fragmented, end-repaired, and polyadenylated before ligation of TruSeq adapters. The adapters contain the index for multiplexing. Fragments containing TruSeq adapters on both ends were selectively enriched with PCR. The quality and quantity of the enriched libraries were validated using Qubit (1.0) Fluorometer and the Bioanalyzer 2100 (Agilent Technologies). The product is a smear with an average fragment size of ∼360 bp. The libraries were normalized to 10 nm in Tris-Cl 10 mm, pH 8.5 with 0.1% Tween 20. The TruSeq SR Cluster Kit v4-cBot-HS or TruSeq PE Cluster Kit v4-cBot-HS (Illumina) was used for cluster generation using 8 pm of pooled normalized libraries on the cBOT. Sequencing was performed on the Illumina HiSeq 4000 single-end 126 bp using the TruSeq SBS Kit v4-HS (Illumina).

The raw reads were first cleaned by removing adapter sequences, trimming low quality ends, and filtering reads with low quality (phred quality < 20) using Trimmomatic (Bolger et al., 2014). Sequence alignment of the resulting high-quality reads to the *Mus musculus* reference genome (build GRCm38) and quantification of gene level expression were performed using RSEM (version 1.2.22) (Li and Dewey, 2011). To detect differentially expressed genes, we applied count-based negative binomial model implemented in the software package EdgeR (R version: 3.2.2, edgeR_3.12.0) (Robinson et al., 2010). The differential expression was assessed using an exact test adapted for overdispersed data. Genes showing altered expression (fold change > 1.2) with adjusted (Benjamini and Hochberg method) *p* < 0.05 (indicated as false discovery rate [FDR]) were considered differentially expressed. Within this set of genes, downregulated and upregulated genes were separately subjected to gene ontology analysis of biological processes using the tool Cytoscape (version 3.5.1) (Shannon et al., 2003) and to prediction of enriched transcription factor binding sites using the tool Homer (version 4.9) (Heinz et al., 2010).

##### Experimental design and statistical analysis.

Data processing and statistical analyses were performed using GraphPad Prism (RRID:SCR_002798, version 7.0a) and Microsoft Excel (version 15.27). Data distribution was assumed to be normal, and variances were assumed to be equal, although this was not formally tested due to low *n* value. Sample sizes were chosen according to sample sizes generally used in the field. The investigators were blinded to the genotypes during analysis of morphological and immunohistochemical data, except for those cases in which mutant mice exhibited an obvious phenotype. No randomization methods were used. Two-tailed unpaired Student's *t* test was used if only two conditions or genotypes were compared. In all other cases, one- or two-way ANOVAs followed by Tukey's, Dunnett's, or Sidak's multiple-comparison tests were used, as indicated in the figure legends. *p* < 0.05 was considered to be statistically significant. No samples or data were omitted during the analyses.

##### Data availability.

RNA sequencing data have been deposited in the GEO database with the accession number GSE108231.

## Results

### mTORC1 is robustly reactivated in dedifferentiating SCs

**Figure 1.**
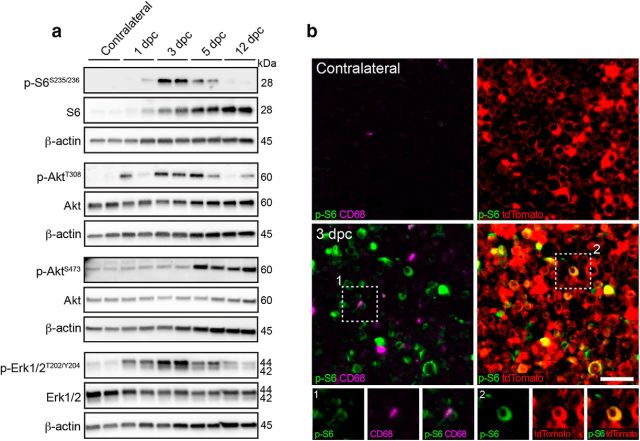
mTORC1 is reactivated in dedifferentiating SCs after nerve injury. ***a***, Western blot analysis of control (Cre-negative, tamoxifen-injected) crushed (distal stump) and contralateral sciatic nerves (*n* = 2 mice per time point). Full-length blots are shown in Figure 1-1 and Figure 1-2. ***b***, Immunostaining for phosphorylated S6 (S235/236) and CD68 of *Mpz*^Cre^*:Rosa26*^tdTomato^ crushed (distal stump) and contralateral sciatic nerves combined with imaging of tdTomato Cre-activity reporter (*n* = 4 mice). Scale bar, 25 μm. Inset 1, An exemplary phospho-S6/CD68 double-positive cell. Inset 2, An example of SCs double-positive for phospho-S6 and tdTomato is magnified.

**Figure 2.**
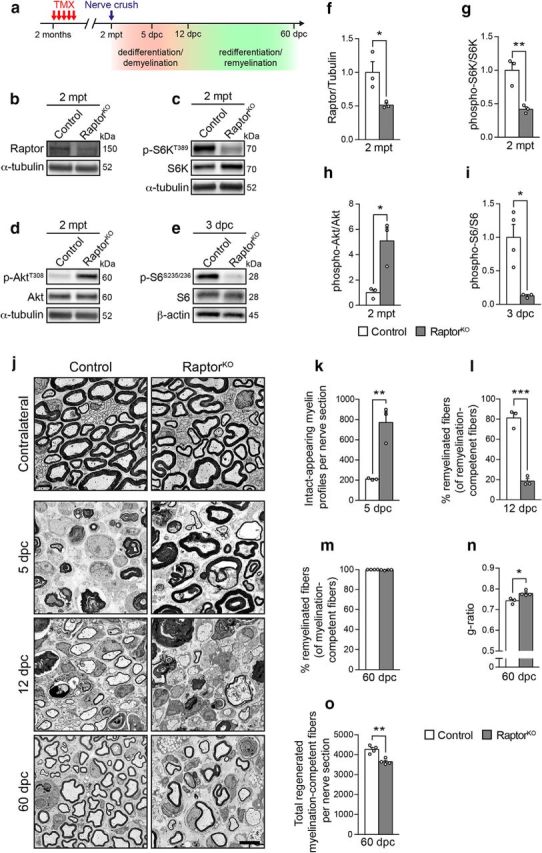
Loss of mTORC1 signaling impairs myelin clearance after nerve injury and delays the subsequent remyelination. ***a***, Outline of the nerve crush injury experiments. TMX, Tamoxifen; mpt, months post tamoxifen. ***b–e***, Western blot analysis of control and RaptorKO uninjured sciatic nerves at 2 mpt (*n* = 3 mice per genotype, *n* = 4 controls and 3 RaptorKOs for the Western blot in ***e***). Full-length blots are shown in Figure 2-1 and Figure 2-2. ***f–i***, Quantifications referring to ***b–e***. Data are expressed as fold change relative to control nerves after normalization to α-tubulin or β-actin (*n* = 3 mice per genotype, *n* = 4 controls and 3 RaptorKOs in ***i***). ***f***, *p* = 0.0385, *t*_(4)_ = 3.036; ***g***, *p* = 0.009, *t*_(4)_ = 4.752; ***h***, *p* = 0.0172, *t*_(4)_ = 3.923; ***i***, *p* = 0.0122, *t*_(5)_ = 3.835 (two-tailed unpaired Student's *t* tests). Bar heights represent mean. Error bars indicate SEM. ***j***, Representative electron micrographs of contralateral and crushed (distal stump) sciatic nerves from control and RaptorKO mice at the indicated time points (*n* = 3 mice per genotype for 5 and 12 dpc, *n* = 4 mice per genotype for 60 dpc). Scale bar, 4 μm. ***k***, Quantification of intact-appearing myelin profiles at 5 dpc in crushed sciatic nerves (distal stump, tibialis fascicle) from control and RaptorKO mice (*n* = 3 mice per genotype, *p* = 0.0059, *t*_(4)_ = 5.354, two-tailed unpaired Student's *t* test). Bar heights represent mean. Error bars indicate SEM. ***l***, Quantification of remyelinated fibers expressed as a percentage of myelination-competent fibers (sorted, and with axons >1 μm in diameter) at 12 dpc (*n* = 3 mice per genotype, *p* = 0.0004, *t*_(4)_ = 10.65, two-tailed unpaired Student's *t* test). Bar heights represent mean. Error bars indicate SEM. ***m***, Quantification of remyelinated fibers expressed as a percentage of myelination-competent fibers (sorted, and with axons >1 μm in diameter) at 60 dpc (*n* = 4 mice per genotype, *p* = 0.086, *t*_(6)_ = 2.052, two-tailed unpaired Student's *t* test). Bar heights represent mean. Error bars indicate SEM. ***n***, Myelin thickness quantified as g-ratio (*n* = 3 control and 4 RaptorKO mice, *p* = 0.0166, *t*_(5)_ = 3.54, two-tailed unpaired Student's *t* test). At least 100 axons per nerve were analyzed (random EM fields). Bar heights represent mean. Error bars indicate SEM. ***o***, Quantification of total regenerated myelination-competent fibers (both myelinated and not, sorted, and with axons >1 μm in diameter) across the entire sciatic nerve section at 60 dpc (*n* = 4 mice per genotype, *p* = 0.0024, *t*_(6)_ = 5.041, two-tailed unpaired Student's *t* test). Bar heights represent mean. Error bars indicate SEM. *p < 0.05, **p < 0.01, ***p < 0.001.

During nerve development, mTORC1 activity is dynamically regulated in relation to the differentiation status of SCs: high before onset of myelination, but lower as SCs start myelinating (Heller et al., 2014; Beirowski et al., 2017; Figlia et al., 2017). In light of these findings, we asked whether, and how, mTORC1 activity varies during dedifferentiation and redifferentiation of SCs. To answer this question, we turned to the nerve crush injury model, in which the sequence of SC dedifferentiation and redifferentiation is well characterized (Stoll et al., 2002; Savastano et al., 2014). We performed unilateral sciatic nerve crush injuries in adult (4-month-old) control mice and assessed the phosphorylation of the ribosomal protein S6 as a measure of mTORC1 activity. We also assessed the activity of two major pathways upstream of mTORC1, PI3K-Akt, and Mek-Erk1/2, by monitoring phosphorylation of Akt and Erk1/2 (Saxton and Sabatini, 2017). In line with previous reports (Heller et al., 2014; Beirowski et al., 2017; Figlia et al., 2017), contralateral uninjured nerves had low (undetectable at the shown exposure time) levels of phospho-S6 and phospho-Akt^T308^, whereas slight phospho-Akt^S473^ and phospho-Erk1/2 signals were visible (Fig. 1*a*; Fig. 1-1; Fig. 1-2). In contrast, upon nerve injury, mTORC1 and both upstream pathways were robustly reactivated. Specifically, phospho-S6, phospho-Akt^T308^, and phospho-Erk1/2 levels were increased in the phase coinciding with SC dedifferentiation from 1 to 5 d after injury (days post crush [dpc]), with a peak at 3 dpc for phospho-S6 and phospho-Erk1/2. Phospho-Akt^S473^ started also to be elevated at 5 dpc. Later on, at 12 dpc, when SCs redifferentiate and start remyelinating, phosphorylation of these proteins decreased to near preinjury levels, with the exception of phospho-Akt^S473^ whose levels remained similarly high also at this time point. Incidentally, we also noticed progressively higher amounts of total S6 protein after nerve injury (Fig. 1*a*; Fig. 1-1), which might reflect increased cellular content of ribosomes.

10.1523/JNEUROSCI.3619-17.2018.f1-1 Figure 1-1 **Full-length western blot images overlaid with the corresponding membranes.** Western blots in Figure 1a. The membrane was cut as indicated by the continuous line and probed with the indicated antibodies. Download Figure 1-1, PDF file

10.1523/JNEUROSCI.3619-17.2018.f1-2 Figure 1-2 **Full-length western blot images overlaid with the corresponding membranes.** Western blots in Figure 1a. The membrane was probed with the indicated antibodies. Download Figure 1-2, PDF file

As nerve injury is accompanied by invasion of inflammatory cells, we then asked to what extent SCs contribute to the high mTORC1 activity detected in whole sciatic nerve lysates. To this end, we stained injured and contralateral nerves for phospho-S6 at 3 dpc, when mTORC1 activity was the highest, using CD68 costaining and the reporter line *Rosa26*^tdTomato^ under *Mpz*^Cre^-driven recombination to mark macrophages and SCs, respectively. As expected, CD68-positive cells were more abundant in injured than in contralateral nerves (Fig. 1*b*). Additionally, while in contralateral nerves most tdTomato-positive SCs displayed the typical crescent-shape staining of myelinating SC cytoplasm, this feature was less prominent in injured nerves, most likely reflecting the dynamic changes that the tissue undergoes upon injury. Consistent with the previous findings, we found strong phospho-S6 signals in injured, but not in contralateral nerves (Fig. 1*b*). The tissue changes associated with injury limited to some extent the precision of further quantitative analyses. However, to provide a rough estimate of the contributions by SCs or macrophages to the high mTORC1 activity detected, we quantified cells double-positive for phospho-S6 and either tdTomato or CD68 in injured nerves, excluding cells double-positive for tdTomato and CD68 because they may represent macrophages containing SC debris. Approximately half of the phospho-S6-positive signals present in injured nerves were also positive for tdTomato (52.65 ± 4.15%, mean ± SEM, *n* = 4 mice), indicating that they belong to SCs or SC-derived structures. A considerably lower percentage (12.2 ± 1.77%, mean ± SEM, *n* = 4 mice) was double-positive for phospho-S6 and the macrophage marker CD68 (Fig. 1*b*). Additionally, phospho-S6-positive macrophages showed generally a weaker phospho-S6 signal compared with SCs.

In sum, mTORC1 is rapidly and robustly reactivated in adult SCs undergoing dedifferentiation, whereas its activity decreases as subsequent remyelination takes place. Together with mTORC1, the PI3K-Akt and Mek-Erk1/2 pathways are also reactivated and follow a similar temporal profile as mTORC1.

### Reactivation of mTORC1 is required for proper myelin clearance upon injury

To investigate the function of mTORC1 reactivation in SCs of injured nerves, we disrupted mTORC1 in adult SCs by deleting the mTORC1-subunit Raptor in an inducible and conditional manner through *Plp1*^CreERT2^-driven recombination of floxed *Rptor* alleles. We induced recombination by injecting Cre-positive and Cre-negative floxed-homozygous mice (*Plp1*^CreERT2^*:Rptor*^flox/flox^ and *Rptor*^flox/flox^, hereafter referred to as RaptorKOs and controls, respectively) with tamoxifen at 2 months of age, when developmental myelination is largely completed (Fig. 2*a*). Two months later, Raptor protein levels were significantly reduced in RaptorKO nerves compared with control nerves (Fig. 2*b*,*f*; Fig. 2-1). Undetectable levels of phospho-S6 in adult uninjured nerves precluded its use to confirm loss of mTORC1 activity. However, phosphorylation of the mTORC1 target S6K (Hay and Sonenberg, 2004) was detectable in control nerves and substantially reduced in RaptorKOs (Fig. 2*c*,*g*; Fig. 2-1). Conversely, Akt was hyperphosphorylated in RaptorKOs (Fig. 2*d*,*h*: Fig. 2-1), indicating loss of the negative feedback loop from mTORC1 to PI3K-Akt (Efeyan and Sabatini, 2010; Norrmén et al., 2014; Figlia et al., 2017).

10.1523/JNEUROSCI.3619-17.2018.f2-1 Figure 2-1 **Full-length western blot images overlaid with the corresponding membranes.** Western blots in Figure 2b-d. The membrane was cut as indicated by the continuous line and probed with the indicated antibodies. Download Figure 2-1, PDF file

10.1523/JNEUROSCI.3619-17.2018.f2-2 Figure 2-2 **Full-length western blot images overlaid with the corresponding membranes.** Western blots in Figure 2e. The membrane was cut as indicated by the continuous line and probed with the indicated antibodies. Note: While detecting the phospho-S6 signal, part of the membrane was covered as indicated by the red line to prevent interference from stronger signals from previous detections. Download Figure 2-2, PDF file

Next, we subjected control and mutant animals to unilateral nerve crush injuries. Consistent with loss of mTORC1 activity, the peak of S6 phosphorylation observed in control conditions at 3 dpc was abolished in RaptorKO-injured nerves (Figs. 1*a*, 2*e*,*i*; Fig. 1-1; Fig. 2-2). Morphologically, no major abnormalities were noticeable in contralateral uninjured mutant nerves compared with controls (Fig. 2*j*). In contrast, while injured control nerves showed advanced demyelination/myelin clearance at 5 dpc, this was substantially impaired in RaptorKOs, as indicated by an almost fourfold increase in intact-appearing myelin profiles (defined as nondiscontinuous and noncollapsed myelin rings) (Fig. 2*k*). At later time points after injury, demyelination/myelin clearance eventually occurred also in RaptorKO nerves (Fig. 2*j*). Presumably as a consequence of the delay in demyelination/myelin clearance, subsequent remyelination was strongly impaired. At 12 dpc, when most fibers were already remyelinated in control nerves, only a minority of myelination-competent fibers was remyelinated in mutant nerves (Fig. 2*l*). Although almost all myelination-competent fibers were remyelinated in both controls and mutants at 60 dpc, with no significant difference between the two genotypes (Fig. 2*m*), mutant nerves displayed thinner myelin as indicated by an overall increase in g-ratio (axon diameter/fiber diameter) (Fig. 2*n*). Additionally, at the same time point, we noticed a mild, but significant, decrease in the total number of regenerated myelination-competent nerve fibers in mutant nerves (Fig. 2*o*).

Together, our data indicate that reactivation of mTORC1 in dedifferentiating SCs is necessary for prompt clearance of myelin in injured nerves and for accurate remyelination of regenerated axons. Furthermore, one interpretation concerning the observation of slightly reduced numbers of regenerated myelination-competent axons in 60 dpc RaptorKO nerves is that mTORC1 activity in SCs might also be subtly required for correct axonal regeneration and/or neuronal survival in our experimental setting.

### Impaired demyelination in RaptorKO nerves is due to defects in SC dedifferentiation and not in the recruitment of phagocytic infiltrating cells

Myelin clearance after injury involves at least two distinct processes: (1) SCs dedifferentiate and degrade their own myelin, for instance, through autophagy and TAM receptor-mediated phagocytosis (Gomez-Sanchez et al., 2015; Jang et al., 2016; Brosius Lutz et al., 2017); and (2) professional phagocytes, including macrophages and neutrophils, are recruited to injured nerves to assist SCs in myelin clearance (Dailey et al., 1998; Klein and Martini, 2016; Lindborg et al., 2017). Additionally, perineurial cells may also contribute, at least in zebrafish (Lewis and Kucenas, 2014). We then tested whether the delayed demyelination of RaptorKO nerves reflects an altered SC dedifferentiation program or altered recruitment and/or function of phagocytic infiltrating cells.

**Figure 3.**
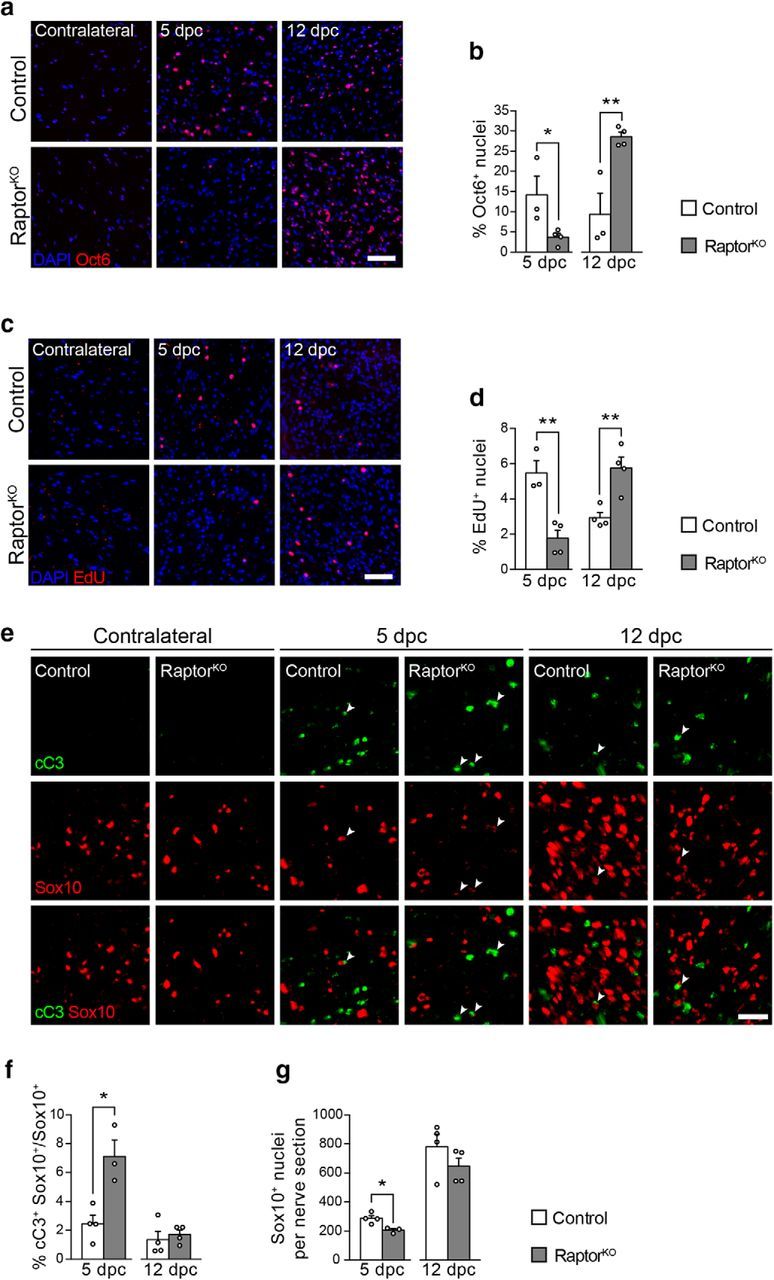
SC dedifferentiation is defective in RaptorKO-injured nerves. ***a***, Oct6 immunostaining of transverse cryosections from control and RaptorKO contralateral and crushed (distal stump) sciatic nerves at 5 and 12 dpc (*n* = 3 controls and 4 RaptorKOs per time point). Scale bar, 50 μm. ***b***, Quantification referring to ***a***. Data are expressed as percentage of Oct6-positive nuclei relative to all DAPI-positive nuclei across the entire sciatic nerve section (*n* = 3 controls and 4 RaptorKOs per time point; 5 dpc, *p* = 0.049, *t*_(5)_ = 2.57; 12 dpc, *p* = 0.008, *t*_(5)_ = 4.2; two-tailed unpaired Student's *t* test). Bar heights represent mean. Error bars indicate SEM. ***c***, EdU staining of transversal cryosections from control and RaptorKO contralateral and crushed (distal stump) sciatic nerves at 5 and 12 dpc (*n* = 3 controls and 4 RaptorKOs for 5 dpc, *n* = 4 mice per genotype for 12 dpc). Scale bar, 50 μm. ***d***, Quantification referring to ***c***. Data are expressed as percentage of EdU-positive nuclei relative to all DAPI-positive nuclei across the entire sciatic nerve section (*n* = 3 controls and 4 RaptorKOs for 5 dpc, *n* = 4 mice per genotype for 12 dpc; 5 dpc, *p* = 0.005, *t*_(5)_ = 4.67; 12 dpc, *p* = 0.007, *t*_(6)_ = 3.99; two-tailed unpaired Student's *t* test). Bar heights represent mean. Error bars indicate SEM. ***e***, cC3 and Sox10 coimmunostaining of transverse cryosections from control and RaptorKO contralateral and crushed (distal stump) sciatic nerves at 5 and 12 dpc (*n* = 3 RaptorKOs at 5 dpc, *n* = 4 controls and RaptorKOs for other conditions). Arrowheads indicate cells double-positive for cC3 and Sox10. Scale bar, 25 μm. ***f***, Quantification referring to ***e***. Data are expressed as percentage of cC3/Sox10 double-positive cells relative to all Sox10-positive SCs across the entire sciatic nerve section (*n* = 3 RaptorKOs at 5 dpc, *n* = 4 controls and RaptorKOs for other conditions; 5 dpc, *p* = 0.01, *t*_(5)_ = 3.98; 12 dpc, *p* = 0.6127, *t*_(6)_ = 0.53; two-tailed unpaired Student's *t* test). Bar heights represent mean. Error bars indicate SEM. ***g***, Quantification referring to ***e***. Data are expressed as number of Sox10-positive cells across the entire sciatic nerve section (*n* = 3 RaptorKOs at 5 dpc, *n* = 4 controls and RaptorKOs for other conditions; 5 dpc, *p* = 0.016, *t*_(5)_ = 3.56; 12 dpc, *p* = 0.25, *t*_(6)_ = 1.27; two-tailed unpaired Student's *t* test). Bar heights represent mean. Error bars indicate SEM. *p < 0.05, **p < 0.01.

To this end, we first assessed the differentiation status of SCs in control and mutant-injured nerves by immunohistochemistry. At 5 dpc, dedifferentiated Oct6-positive SCs were significantly reduced in RaptorKO nerves compared with control nerves (Fig. 3*a*,*b*). Conversely, at 12 dpc, more Oct6-positive cells were present in mutant nerves compared with controls, likely reflecting the delayed onset of remyelination observed in these animals. Although proliferation of SCs in the distal nerve stump is not essential for successful nerve regeneration (Yang et al., 2008), it is an obligatory feature and reflects the differentiation status of the cells. We found that the observed changes in Oct6-positive cells were closely matched with altered numbers of EdU-positive cells (Fig. 3*c*,*d*), reminiscent of the proposed positive effect of mTORC1 activity on SC proliferation in development (Beirowski et al., 2017). Conversely, SC apoptosis, assessed by cleaved-caspase 3 positivity, was increased in injured mutant nerves at 5 dpc compared with control nerves, but not at 12 dpc (Fig. 3*e*,*f*). Together with the observed reduced proliferation at 5 dpc (Fig. 3*c*,*d*), this effect is likely to contribute to the slight reduction in the number of SCs in injured mutant nerves at the same time point (Fig. 3*g*). As expected, the contralateral uninjured nerves contained only rare Oct6- or EdU-positive cells and no detectable cleaved-caspase 3-positive cells in either controls or RaptorKOs (Fig. 3*a*,*c*,*e*).

**Figure 4.**
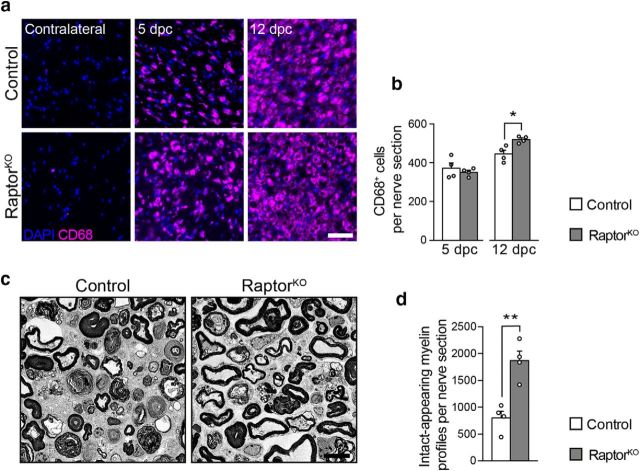
Reduced myelin clearance in RaptorKO nerves does not depend on altered macrophage recruitment. ***a***, CD68 immunostaining of transverse cryosections from control and RaptorKO contralateral and crushed (distal stump) sciatic nerves at 5 and 12 dpc (*n* = 4 mice per genotype). Scale bar, 50 μm. ***b***, Quantification of macrophages in the 5 and 12 dpc crushed nerves in ***a***. Data are expressed as number of CD68-positive cells per sciatic nerve cross section (*n* = 4 mice per genotype and time point; 5 dpc, *p* = 0.49, *t*_(6)_ = 0.72; 12 dpc, *p* = 0.013, *t*_(6)_ = 3.46; two-tailed unpaired Student's *t* test). Bar heights represent mean. Error bars indicate SEM. ***c***, Representative electron micrographs of control and RaptorKO uninjured sciatic nerves, explanted and kept in culture for 7 d (*n* = 4 mice per genotype). Scale bar, 4 μm. ***d***, Quantification referring to ***c***. Data are expressed as number of intact-appearing myelin profiles per sciatic nerve cross section (*n* = 4 mice per genotype, *p* = 0.0027, *t*_(6)_ = 4.915, two-tailed unpaired Student's *t* test). Bar heights represent mean. Error bars indicate SEM. *p < 0.05, **p < 0.01.

Next, we evaluated the presence and abundance of macrophages at 5 and 12 dpc by immunohistochemistry. In both controls and mutants, nerve injury was followed by a marked invasion of CD68-positive macrophages compared with uninjured contralateral nerves, with no significant difference between the two genotypes at 5 dpc, and a mild, albeit significant increase in the number of macrophages in RaptorKOs compared with controls at 12 dpc (Fig. 4*a*,*b*). This indicates that the recruitment of macrophages after injury is not detectably impaired after loss of mTORC1 function in SCs.

Finally, we reasoned that, if the impaired myelin clearance of RaptorKOs is primarily a consequence of altered SC dedifferentiation, we should observe a similar phenotype also in the absence of blood-derived inflammatory cells. To model this situation, we explanted segments of uninjured nerves from controls and mutants, cultured them for 7 d to allow axons to degenerate and SCs to dedifferentiate, and quantified the abundance of intact-appearing myelin profiles. Also in this setting, mutant nerves contained more intact-appearing myelin profiles than control nerves, although the difference between the two genotypes was less pronounced than *in vivo* (Fig. 4*c*,*d*).

Collectively, our results indicate that the delayed myelin clearance in RaptorKO nerves reflects a general defect in SC dedifferentiation rather than a failure in the recruitment of professional phagocytic cells. Thus, reactivation of mTORC1 upon injury is a prerequisite for efficient SC dedifferentiation.

### Mek-Erk signaling is not altered in RaptorKO nerves

**Figure 5.**
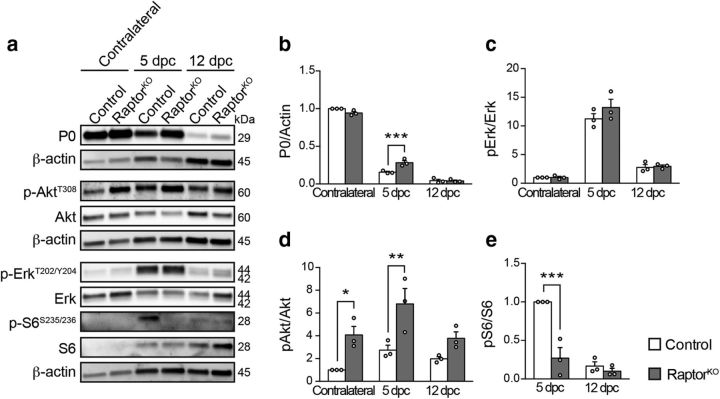
Analysis of pathways upstream of mTORC1 after nerve injury. ***a***, Western blot analysis of control and RaptorKO contralateral and crushed (distal stump) sciatic nerves at 5 and 12 dpc (*n* = 3 mice per genotype and time point). Full-length blots are shown in Figure 5-1. ***b–e***, Quantifications referring to ***a***. Data are expressed as fold change relative to contralateral control nerves (***b–d***) or 5 dpc control nerves (***e***) (*n* = 3 mice per genotype and time point). ***b***, *p* = 0.0004, *F*_(2,12)_ = 16.01, *p*_5dpc_ = 0.0005; ***c***, *p* = 0.365, *F*_(2,12)_ = 1.097; ***d***, *p* = 0.3121, *F*_(2,12)_ = 1.285, *p*_contralateral_ = 0.0278, *p*_5dpc_ = 0.0046; ***e***, *p* = 0.0024, *F*_(1,8)_ = 18.98, *p*_5dpc_ = 0.0003 (two-way ANOVA with Sidak's multiple-comparison test). Bar heights represent mean. Error bars indicate SEM. *p < 0.05, **p < 0.01, ***p < 0.001.

Although loss of mTORC1 activity substantially delayed SC dedifferentiation after injury, other mechanisms appear to compensate for its absence in the longer term. mTORC1 signaling has been shown in some cell systems to limit Erk activation through negative feedback loops analogous to those described for PI3K-Akt (Carracedo et al., 2008). As constitutive activation of the Mek-Erk pathway can be sufficient to drive SC dedifferentiation (Napoli et al., 2012), we considered the possibility that the defective SC dedifferentiation in RaptorKO nerves could eventually recover through increased Erk activity caused by loss of the mTORC1-dependent feedback loops. A similar compensatory mechanism involving the Mek-Erk pathway has been previously invoked for oligodendrocyte myelination upon ablation of Raptor (Bercury et al., 2014). To test this hypothesis, we assessed the phosphorylation levels of Erk, together with Akt and S6, in control and mutant nerves. We analyzed contralateral uninjured nerves and injured nerves at 5 dpc, when RaptorKO nerves still contained substantial amounts of myelin, and at 12 dpc, when myelin has been cleared and remyelination has started (Figs. 2*j*,*k*, 5*a*,*b*; Fig. 5-1). Consistent with the previous results (Fig. 1*a*; Fig. 1-1), phospho-S6 levels were undetectable in uninjured nerves of either genotype, whereas they robustly increased at 5 dpc in controls, but not in mutants (Fig. 5*a*,*e*; Fig. 5-1). However, at 12 dpc, no significant difference in S6 phosphorylation was detectable anymore between the two genotypes. The progressive reduction of S6 phosphorylation in controls combined with an increase in macrophages may contribute to this finding (Fig. 4*a*). Neither in contralateral nor in injured nerves, at either time point, could we detect differences in Erk phosphorylation between controls and mutants (Fig. 5*a*,*c*; Fig. 5-1). In contrast, Akt phosphorylation at T308 was, as expected, significantly increased compared with controls in contralateral and injured RaptorKO nerves at 5 dpc, with a tendency also at 12 dpc (Fig. 5*a*,*d*; Fig. 5-1).

10.1523/JNEUROSCI.3619-17.2018.f5-1 Figure 5-1 **Full-length western blot images overlaid with the corresponding membranes.** Western blots in Figure 5a. The membrane was cut as indicated by the continuous line and probed with the indicated antibodies. Download Figure 5-1, PDF file

Based on these findings, we conclude that a compensatory role for Erk signaling when mTORC1 is disrupted in SCs of injured nerves is unlikely.

### mTORC1 signaling is necessary for c-Jun upregulation upon injury

**Figure 6.**
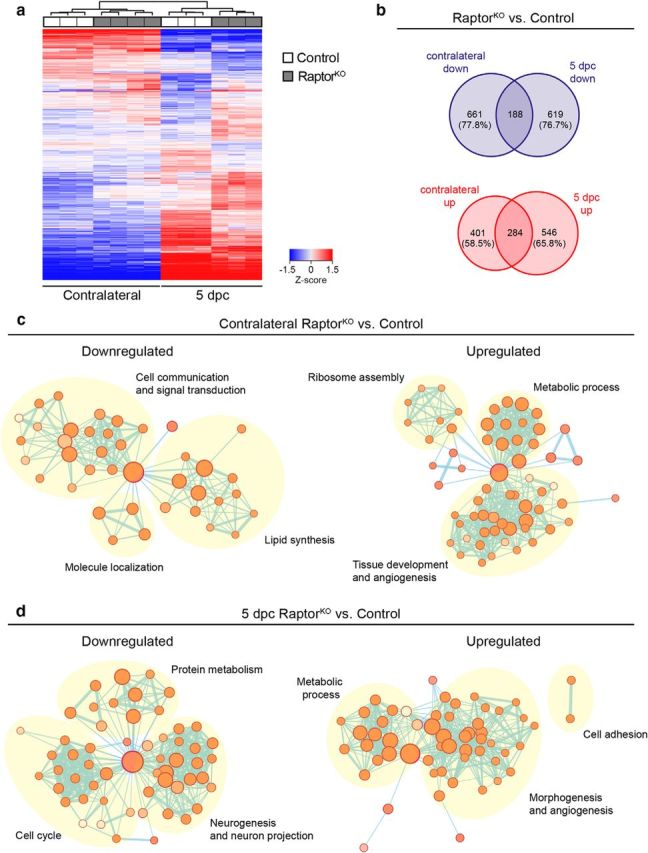
Transcriptome profiles of control and RaptorKO injured and contralateral nerves at 5 dpc. ***a***, Heatmap and unbiased cluster analysis of transcriptome profiles from control and RaptorKO contralateral and crushed (distal stump) sciatic nerves at 5 dpc (*n* = 4 mice for RaptorKO contralateral nerves, *n* = 3 for the other conditions). ***b***, Venn diagrams of differentially expressed genes (FDR < 0.05, fold change > 1.2) from RaptorKO contralateral and crushed nerves compared with contralateral and crushed control nerves, respectively. The list of all genes used for this analysis is in Figure 6-1. The percentage of genes differentially expressed in contralateral but not in crushed nerves and vice versa is indicated. ***c***, ***d***, Gene ontology analysis of differentially expressed genes in RaptorKO contralateral and crushed nerves compared with contralateral and crushed control nerves, respectively, expressed as enrichment maps. Nodes indicate sets of genes belonging to similar gene ontology categories, whereas edges indicate overlap between two different gene sets. The list of all genes used for this analysis is in Figure 6-1.

To investigate the mechanisms whereby mTORC1 affects SC dedifferentiation, we analyzed the transcriptomes of control and RaptorKO-injured nerves at 5 dpc, together with the corresponding contralateral nerves. Heatmap and unbiased hierarchical cluster analysis showed close similarity between different replicates of the same genotype and/or condition (Fig. 6*a*), thus validating the subsequent analyses. We identified 807 downregulated and 830 upregulated genes in RaptorKO-injured nerves compared with injured control nerves, and 849 downregulated and 685 upregulated genes in RaptorKO contralateral nerves compared with contralateral control nerves (FDR < 0.05, fold change > 1.2). Interestingly, most of the differentially expressed genes were not shared between mutant injured and contralateral nerves (Fig. 6*b*; Fig. 6-1), suggesting that the impaired dedifferentiation in mutants originates from a specific failure in the SC response to nerve injury, rather than from preexisting molecular changes. Gene ontology analysis of the differentially expressed genes revealed for the contralateral mutant nerve an enrichment in similar terms as those previously reported for developmental knock-outs of Raptor in SCs (Figlia et al., 2017), such as lipid synthesis among the downregulated genes and ribosome assembly among the upregulated ones (Fig. 6*c*; Fig. 6-1. In injured mutant nerves, protein metabolism, neurogenesis/neuron projection, and cell cycle-related terms were enriched among the downregulated genes, in line with the lowered proliferation rate in RaptorKO nerves at 5 dpc (Fig. 3*c*,*d*). Interestingly, both in mutant injured and mutant contralateral nerves, upregulated genes were also enriched in angiogenesis-related terms (Fig. 6*c*,*d*; Fig. 6-1).

**Figure 7.**
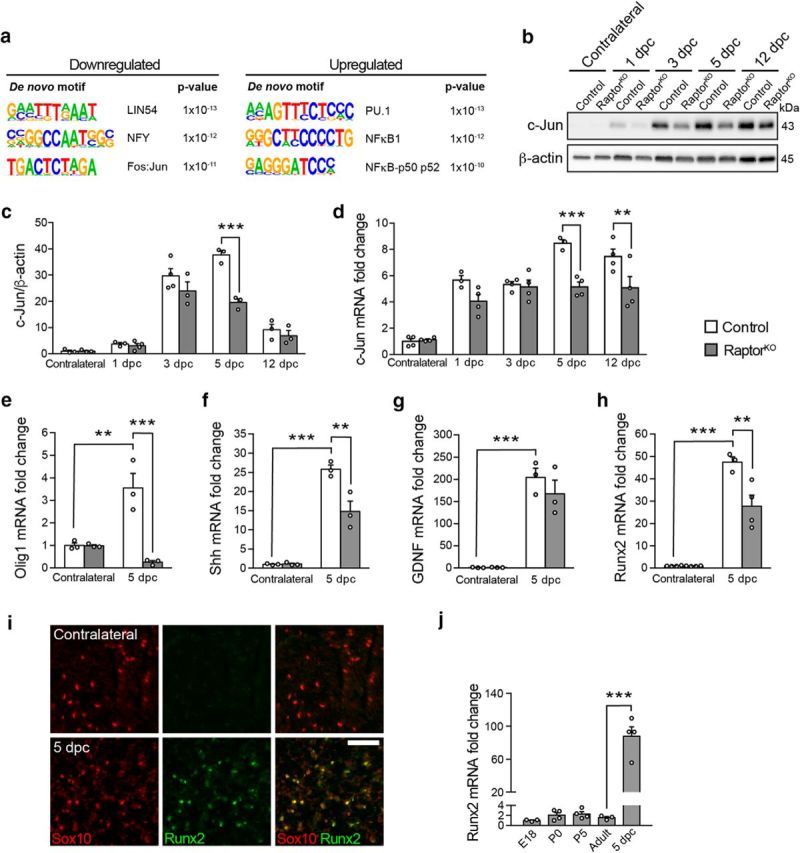
Loss of mTORC1 impedes normal upregulation of c-Jun and its downstream targets upon nerve injury. ***a***, Top motifs enriched in the promoter sequences of downregulated and upregulated genes in RaptorKO crushed nerves at 5 dpc compared with crushed control nerves. The transcription factors corresponding to the enriched motifs are indicated. The list of all genes used for this analysis is in Figure 6-1. ***b***, Western blot analysis of c-Jun in contralateral and crushed (distal stump) control and RaptorKO sciatic nerves at various time points (*n* = 3 mice per condition, except for 1 dpc RaptorKO and 3 dpc control, for which *n* = 4). Full-length blots are shown in Figure 7-1. ***c***, Quantification referring to ***b***. Data are expressed as fold change relative to control contralateral nerves after normalization to β-actin (*n* = 3 mice per condition, except for 1 dpc RaptorKO and 3 dpc control, for which *n* = 4, *p* = 0.0007, *F*_(4,22)_ = 7.336, two-way ANOVA with Sidak's multiple-comparison test: 5 dpc, *p* < 0.0001). Bar heights represent mean. Error bars indicate SEM. ***d***, qRT-PCR analysis of c-Jun expression in RaptorKO and control contralateral and crushed (distal stump) sciatic nerves at various time points. Data are expressed as fold change relative to control contralateral nerves after normalization to GAPDH (*n* = 4 mice per condition, except for 1 and 5 dpc control crushed nerves, for which *n* = 3, *p* = 0.0041, *F*_(4,28)_ = 4.873, two-way ANOVA with Sidak's multiple-comparison test: 5 dpc, *p* = 0.0002; 12 dpc, *p* = 0.0043). Bar heights represent mean. Error bars indicate SEM. ***e–h***, qRT-PCR analysis of selected repair-cell gene expression in RaptorKO and control contralateral and crushed (distal stump) sciatic nerves at 5 dpc. Data are expressed as fold change relative to control contralateral nerves after normalization to GAPDH (*n* = 3 mice per condition, except for contralateral control, contralateral RaptorKO, and crushed RaptorKO nerves in ***h***, for which *n* = 4). ***e***, *p* = 0.0011, *F*_(1,8)_ = 24.4; ***f***, *p* = 0.0054, *F*_(1,8)_ = 14.33; ***g***, *p* = 0.3493, *F*_(1,8)_ = 0.9881; ***h***, *p* = 0.005, *F*_(1,11)_ = 12.2 (two-way ANOVA with Sidak's multiple-comparison test). **e**, *p*_control contralateral vs crushed_ = 0.0037, *p*_crushed control vs KO_ = 0.0007; ***f***, *p*_control contralateral vs crushed_ < 0.0001, *p*_crushed control vs KO_ = 0.0044; ***g***, *p*_control contralateral vs crushed_ = 0.0003; ***h***, *p*_control contralateral vs crushed_ < 0.0001, *p*_crushed control vs KO_ = 0.0034. The qRT-PCR results closely match the RNA sequencing results, with the exception of Runx2, which was reduced only in tendency in the transcriptomic analysis. Bar heights represent mean. Error bars indicate SEM. ***i***, Sox10 and Runx2 immunostaining of transverse cryosections from crushed (distal stump) and contralateral control nerves at 5 dpc (*n* = 3 contralateral and 4 crushed nerves). Scale bar, 50 μm. ***j***, qRT-PCR analysis of Runx2 expression in sciatic nerves from control mice of different ages: embryonic day (E) 18, postnatal day (P) 0, P5, adult (4-month-old uninjured nerves), and 5 dpc (4-month-old crushed nerves). Data are expressed as fold change relative to E18 nerves after normalization to GAPDH (*n* = 4 mice per time point, except for E18 and adult mice, for which *n* = 3, *p* < 0.0001, *F*_(4,13)_ = 47.76, one-way ANOVA with Sidak's multiple-comparison test: *p*_adult vs 5 dpc_ < 0.0001). Bar heights represent mean. Error bars indicate SEM. **p < 0.01, ***p < 0.001.

10.1523/JNEUROSCI.3619-17.2018.f6-1 Figure 6-1 **Transcriptome analysis.** Lists of all expressed genes (defined as number of reads greater or equal to 50 in more than one sample) from RNA-sequencing analysis of contralateral and crushed (distal stump) sciatic nerves from controls and RaptorKOs at 5 dpc. The significantly (FDR < 0.05, fold change >1.2) downregulated and upregulated genes used for the analyses in Figure 6b-d and Figure 7a are reported separately. Download Figure 6-1, XLSX file

To predict which transcription factors might be responsible for the observed transcriptional changes, we then analyzed bioinformatically the promoter sequences of the genes differentially expressed in RaptorKO-injured nerves versus injured control nerves in search of enriched transcription-factor binding sites (Fig. 7*a*; Fig. 6-1). Using Homer Motif Analysis (http://homer.ucsd.edu/homer/motif/), we found motifs closely matching the binding sites of Lin54, NFY, and the dimer Jun-Fos among the top motifs enriched in the promoters of downregulated genes, and among the upregulated genes, those of PU.1 and the NFκB family. c-Jun is a major transcription factor strongly upregulated by dedifferentiating SCs and with a central role in coordinating the events after nerve injury (Parkinson et al., 2008; Arthur-Farraj et al., 2012; Fontana et al., 2012). Many features observed in injured c-Jun-knock-out nerves are reminiscent of RaptorKO nerves, including defective SC dedifferentiation and myelin clearance. Thus, we focused on this transcription factor as a possible link between mTORC1 reactivation and SC dedifferentiation. Consistent with previous reports (Parkinson et al., 2008; Arthur-Farraj et al., 2017), expression of c-Jun was strongly increased in injured control nerves compared with contralateral nerves, both at the protein and mRNA levels (Fig. 7*b–d*; Fig. 7-1). In contrast, injured RaptorKO nerves showed a less pronounced upregulation of c-Jun, which was significantly lower than in injured controls at the protein level at 5 dpc and at the mRNA level at both 5 and 12 dpc.

10.1523/JNEUROSCI.3619-17.2018.f7-1 Figure 7-1 **Full-length western blot images overlaid with the corresponding membranes.** Western blots in Figure 7b. The membrane was cut as indicated by the continuous line and probed with the indicated antibodies. Download Figure 7-1, PDF file

The SC response to nerve injury consists not only of dedifferentiation, but also of the novel expression of a set of genes not expressed earlier, such as *Olig1*, *Shh*, and *Gdnf* (Arthur-Farraj et al., 2012). Importantly, many of the repair-SC specific genes are supposed to be under the transcriptional control of c-Jun (Arthur-Farraj et al., 2012, 2017; Fontana et al., 2012; Hung et al., 2015). Thus, to assess whether not only dedifferentiation, but also generation of repair SCs was affected by loss of mTORC1 function, and to assess the potential functional impact of the observed reduction in c-Jun, we analyzed the mRNA levels of Olig1, Shh, and GDNF by qPCR. As expected, Olig1, Shh, and GDNF mRNA levels were strongly elevated in injured control nerves compared with contralateral nerves at 5 dpc (Fig. 7*e–g*). At the same time point, Olig1 and Shh levels were substantially lower in injured RaptorKO nerves compared with injured control nerves, whereas GDNF showed no significant change. The gene encoding the transcription factor Runx2 is a recently characterized c-Jun transcriptional target in SCs (Hung et al., 2015) and is upregulated early upon injury with similar kinetics as c-Jun (Arthur-Farraj et al., 2017). These findings suggested that it might represent another repair-SC gene. Consistent with a c-Jun-dependent regulation, we found a marked reduction in Runx2 mRNA levels in RaptorKO-injured nerves compared with injured control nerves at 5 dpc (Fig. 7*h*). In agreement with a previous report (Hung et al., 2015), Runx2 was undetectable in contralateral control nerves by immunohistochemical analysis but abundantly present in injured nerves at 5 dpc (Fig. 7*i*). Moreover, it almost completely colocalized with the SC-marker Sox10 (98 ± 0.1%, mean ± SEM, *n* = 4 mice), indicating that dedifferentiating SCs are the main cellular source of Runx2 in nerves. To provide further evidence that *Runx2* is a *bona fide* repair-SC gene, we then compared its expression across various time points from embryonic day 18 (E18) to adult injured and uninjured nerves at 5 dpc. Indeed, Runx2 mRNA levels were low and did not change much throughout development and into adulthood, although they increased massively in injured nerves (Fig. 7*j*).

In sum, our results indicate that normal upregulation of c-Jun and of downstream repair-SC genes, including *Runx2*, in dedifferentiating SCs requires reactivation of mTORC1. Based on the well-established function of c-Jun in the SC response to injury, these data further suggest that the impaired dedifferentiation of RaptorKO SCs may derive from insufficient upregulation of c-Jun.

### mTORC1 promotes c-Jun translation

**Figure 8.**
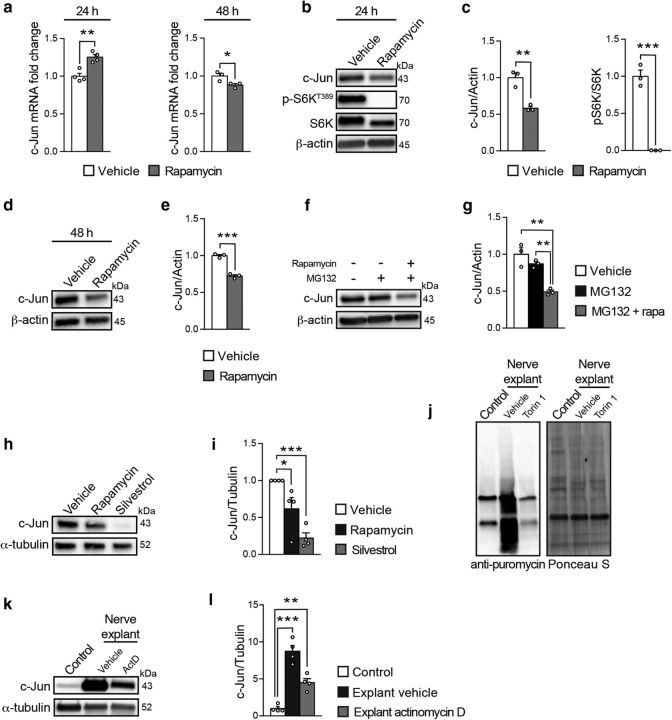
mTORC1 regulates c-Jun by promoting its translation. ***a***, qRT-PCR analysis of c-Jun expression in rSCs treated for 24 or 48 h with vehicle or rapamycin (50 nm) under growth conditions. Data are expressed as fold change relative to vehicle-treated cells after normalization to GAPDH (*n* = 4 biological replicates per condition at 24 h, *n* = 3 biological replicates per condition at 48 h; 24 h, *p* = 0.0024, *t*_(6)_ = 5.011; 48 h, *p* = 0.0437, *t*_(4)_ = 2.91; two-tailed unpaired Student's *t* test). The experiment at 24 h was performed 4 times, and one representative experiment is shown. The experiment at 48 h was performed once with three independent SC preparations. Bar heights represent mean. Error bars indicate SEM. ***b***, Western blot analysis of c-Jun in rSCs treated for 24 h with vehicle or rapamycin (50 nm) under growth conditions (*n* = 3 biological replicates per condition). The experiment was performed 3 times, and one representative experiment is shown. Full-length blots are shown in Figure 8-1. ***c***, Quantification referring to ***b***. Data are expressed as fold change relative to vehicle-treated cells after normalization to β-actin (*n* = 3 biological replicates per condition; c-Jun/actin, *p* = 0.0036, *t*_(4)_ = 6.102; pS6K/S6K, *p* = 0.0003, *t*_(4)_ = 11.98; two-tailed unpaired Student's *t* test). Bar heights represent mean. Error bars indicate SEM. ***d***, Western blot analysis of c-Jun in rSCs treated for 48 h with vehicle or rapamycin (50 nm) under growth conditions (*n* = 3 biological replicates per condition). The experiment was performed once with three independent SC preparations. Full-length blots are shown in Figure 8-2. ***e***, Quantification referring to ***d***. Data are expressed as fold change relative to vehicle-treated cells after normalization to β-actin (*n* = 3 biological replicates per condition, *p* = 0.0003, *t*_(4)_ = 11.86, two-tailed unpaired Student's *t* test). Bar heights represent mean. Error bars indicate SEM. ***f***, Western blot analysis of c-Jun in rSCs treated for 24 h with vehicle, MG132 (5 μm), and/or rapamycin (50 nm) under growth conditions (*n* = 3 biological replicates per condition). The experiment was performed 3 times, and one representative experiment is shown. Full-length blots are shown in Figure 8-3. ***g***, Quantification referring to ***f***. Data are expressed as fold change relative to vehicle-treated cells after normalization to β-actin (*n* = 3 biological replicates per condition, *p* = 0.0016, *F*_(2,6)_ = 22.48, one-way ANOVA with Tukey's multiple-comparison test: *p*_control vs MG132 + rapa_ = 0.0016, *p*_MG132 vs MG132 + rapa_ = 0.0071). Bar heights represent mean. Error bars indicate SEM. ***h***, Western blot analysis of c-Jun in rSCs treated for 48 h with vehicle, rapamycin (50 nm), or silvestrol (25 nm) under growth conditions (*n* = 4 biological replicates per condition). The experiment was performed 4 times, and one representative experiment is shown. Full-length blots are shown in Figure 8-4. ***i***, Quantification referring to ***h***. Data are expressed as fold change relative to vehicle-treated cells after normalization to α-tubulin (*n* = 4 biological replicates per condition, *p* = 0.0011, *F*_(2,9)_ = 15.96, one-way ANOVA with Dunnett's multiple-comparison test: *p*_vehicle vs rapa_ = 0.0376, *p*_vehicle vs silvestrol_ = 0.0006). Bar heights represent mean. Error bars indicate SEM. ***j***, Puromycin incorporation-based assay to evaluate protein synthesis in nerve explants from wild-type mice cultured for 24 h in the presence of vehicle or Torin 1 (250 nm) and compared with wild-type nerves collected immediately after 30 min incubation with puromycin (control) (*n* = 4 mice per condition). Ponceau S staining shows equal protein loading. The experiment was performed twice with independent samples (*n* = 7 mice per condition in total). A representative blot is shown. Full-length blots are shown in Figure 8-5. ***k***, Western blot analysis of c-Jun in nerve explants from wild-type mice cultured for 24 h in the presence of vehicle or actinomycin D (5 μg/ml) and compared with wild-type nerves collected immediately (control) (*n* = 4 mice per condition). The experiment was performed twice with independent samples. A representative blot is shown. Full-length blots are shown in Figure 8-6. ***l***, Quantification referring to ***k***. Data are expressed as fold change relative to control nerves after normalization to α-tubulin (*n* = 4 mice per condition, *p* < 0.0001, *F*_(2,9)_ = 46.39, one-way ANOVA with Dunnett's multiple-comparison test: *p*_control vs vehicle_ = 0.0001, *p*_control vs actD_ = 0.0034). Bar heights represent mean. Error bars indicate SEM. *p < 0.05, **p < 0.01, ***p < 0.001.

Given the heterogeneous cellular composition of injured nerves and the complex interactions between its different cell types, we turned to established rSC culture experiments to study in more detail the regulatory relationship between mTORC1 and c-Jun. Acute inhibition of mTORC1 with rapamycin led to a biphasic response in c-Jun mRNA levels, including a mild increase at 24 h and a slight decrease at 48 h (Fig. 8*a*). In contrast, under the same conditions, protein levels of c-Jun were substantially reduced already after 24 h of rapamycin treatment without further detectable changes after 48 h (Fig. 8*b–e*; Fig. 8-1; Fig. 8-2). Together with the *in vivo* data (Fig. 7*c*,*d*), these results suggest that, in addition to a possible transcriptional control, mTORC1 regulates c-Jun expression also post-transcriptionally, conceivably at the level of translation and/or protein stability. To exclude that the reduction in c-Jun protein levels was caused by increased degradation, we then blocked pharmacologically proteasome-dependent degradation with the drug MG132. Consistent with a major regulation at the level of translation, inhibition of mTORC1 with rapamycin reduced c-Jun protein expression also when proteasome-dependent degradation was blocked (Fig. 8*f*,*g*; Fig. 8-3).

10.1523/JNEUROSCI.3619-17.2018.f8-1 Figure 8-1 **Full-length western blot images overlaid with the corresponding membranes.** Western blots in Figure 8b. The membrane was cut as indicated by the continuous line and probed with the indicated antibodies. X’s indicate unrelated samples. Download Figure 8-1, PDF file

10.1523/JNEUROSCI.3619-17.2018.f8-2 Figure 8-2 **Full-length western blot images overlaid with the corresponding membranes.** Western blots in Figure 8d. The membrane was probed with the indicated antibodies. X’s indicate unrelated samples. Download Figure 8-2, PDF file

10.1523/JNEUROSCI.3619-17.2018.f8-3 Figure 8-3 **Full-length western blot images overlaid with the corresponding membranes.** Western blots in Figure 8f. The membrane was probed with the indicated antibodies. Download Figure 8-3, PDF file

10.1523/JNEUROSCI.3619-17.2018.f8-4 Figure 8-4 **Full-length western blot images overlaid with the corresponding membranes.** Western blots in Figure 8h. The membrane was probed with the indicated antibodies. X’s indicate unrelated samples. Download Figure 8-4, PDF file

10.1523/JNEUROSCI.3619-17.2018.f8-5 Figure 8-5 **Full-length western blot images overlaid with the corresponding membranes.** Western blots in Figure 8j. The membrane was probed with the indicated antibodies or stained with Ponceau S as loading control. Download Figure 8-5, PDF file

10.1523/JNEUROSCI.3619-17.2018.f8-6 Figure 8-6 **Full-length western blot images overlaid with the corresponding membranes.** Western blots in Figure 8k. The membrane was probed with the indicated antibodies. Download Figure 8-6, PDF file

Control of mRNA translation is a well-established function of the mTORC1 pathway (Ma and Blenis, 2009). By inhibiting the 4E-binding proteins and activating S6Ks, mTORC1 promotes the assembly and function of the eIF4F complex. This complex binds mRNA caps, facilitates the recruitment of the 40S ribosomal subunit, and unwinds local secondary structures in the 5′-UTR through its helicase subunit eIF4A (Thoreen, 2013). To further corroborate the translational control of c-Jun by mTORC1 and to assess whether the eIF4F complex is involved, we treated rSCs with silvestrol, a drug selectively inhibiting the eIF4A subunit of eIF4F (Chu et al., 2016). Consistent with our hypothesis, silvestrol-treated cells showed profoundly reduced levels of c-Jun compared with vehicle-treated cells, and also lower levels compared with rapamycin-treated cells (Fig. 8*h*,*i*; Fig. 8-4).

In light of these findings, reactivation of mTORC1 in dedifferentiating SCs might support the accumulation of c-Jun, and potentially other proteins, also by enhancing the translation of their mRNAs. If correct, nerve injury is expected to cause a general increase in protein synthesis in an mTORC1-dependent manner. To test this hypothesis, we evaluated the rate of protein synthesis using a puromycin-incorporation assay (Schmidt et al., 2009) in explanted wild-type nerves incubated for 24 h with vehicle or with the mTOR inhibitor Torin 1. This ATP-competitive drug was chosen instead of rapamycin because phosphorylation of certain mTORC1 targets with a role in protein synthesis is reportedly resistant to rapamycin (Thoreen et al., 2009). Demyelinating vehicle-treated nerve explants showed a marked elevation in protein synthesis compared with control nondemyelinating nerves (collected immediately after dissection) (Fig. 8*j*; Fig. 8-5), and this effect could be substantially attenuated by mTORC1 inhibition with Torin 1. Finally, to confirm that translational control of c-Jun is another layer of regulation in addition to transcriptional control, we compared c-Jun protein levels in control nerves (collected immediately after dissection) and demyelinating nerve explants treated with vehicle or the drug actinomycin D for 24 h to block mRNA transcription. As expected, demyelinating vehicle-treated nerve explants showed a robust increase in c-Jun protein compared with control nerves (Fig. 8*k*,*l*; Fig. 8-6). Although blockage of mRNA transcription substantially reduced this c-Jun upregulation, we still observed an approximately fivefold increase compared with control nerves (Fig. 8*k*,*l*; Fig. 8-6), suggesting that nontranscriptional processes, such as more mRNA translation, are also involved in c-Jun upregulation upon injury. Consistent with this interpretation, we noticed that, whereas c-Jun mRNA levels increase 8–10 times upon injury, c-Jun protein levels increase almost up to 40 times (Fig. 7*c*,*d*).

In sum, our results indicate that mTORC1 promotes c-Jun translation possibly via the eIF4A-containing eIF4F complex, and further suggest that mTORC1-dependent translation of c-Jun is an additional layer of regulation in addition to transcriptional control.

## Discussion

SCs are highly plastic cells and the signals underlying their plasticity are of utmost biological and medical interest. Here, we show that reactivation of the mTORC1 pathway is a key event for SC dedifferentiation. While normal adult SCs exhibit very low mTORC1 activity, this pathway is strongly reactivated in dedifferentiating SCs after injury. Conversely, as remyelination starts, mTORC1 activity declines. This transient but robust mTORC1 reactivation is required for myelin clearance/demyelination and, indirectly, for subsequent remyelination, without obvious defects in the inflammatory response. mTORC1 regulates autophagy (Ben-Sahra and Manning, 2017), and a particular form of autophagy (myelinophagy) in SCs contributes to myelin clearance (Gomez-Sanchez et al., 2015; Jang et al., 2016). A primary defect in myelinophagy is, however, unlikely to account for the impaired myelin clearance upon loss of mTORC1 signaling: (1) mTORC1 suppresses autophagy, thus regular autophagy should be higher, rather than lower; and (2) myelinophagy was shown to occur independently of mTORC1 activity (Gomez-Sanchez et al., 2015). Instead, we found that reduced myelin clearance upon loss of mTORC1 signaling was accompanied by impaired SC dedifferentiation, as indicated by delayed proliferation and aberrant appearance of Oct6-positive cells.

**Figure 9.**
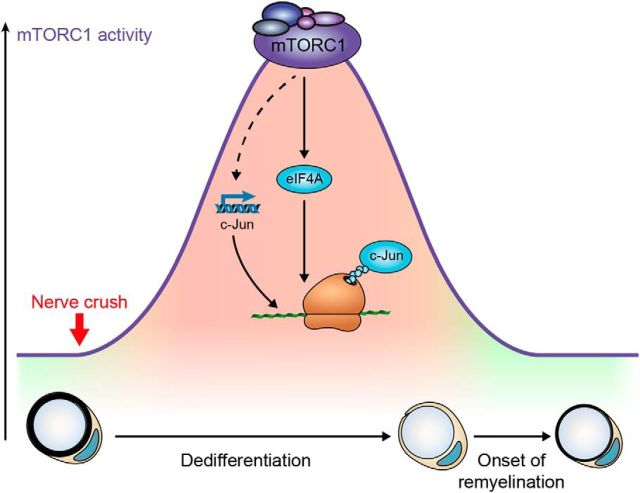
Model of mTORC1 function in SCs after nerve injury. Transient reactivation of mTORC1 in injured nerves promotes c-Jun elevation by transcriptional and post-transcriptional regulation to ensure timely dedifferentiation of SCs.

These results complement and extend previous data on nerve regeneration under conditions of mTORC1 hyperactivation (Figlia et al., 2017). Deletion of TSC1 or PTEN to hyperactivate mTORC1 using a comparable approach as here did not significantly affect myelin clearance or SC dedifferentiation. Thus, although mTORC1 is indispensable for these processes, an additional increase in mTORC1 activity is not able to enhance or accelerate them, suggesting that the mTORC1-dependent molecular machinery for SC dedifferentiation is already maximally active in normal conditions. In contrast, hyperactivation of mTORC1 substantially delayed the onset of remyelination (Figlia et al., 2017). In combination with the present results, the latter observation supports a model in which robust reactivation of mTORC1 upon injury is necessary for SC dedifferentiation, whereas the subsequent decline in mTORC1 activity as SCs redifferentiate is necessary for the onset of remyelination (Fig. 9).

This model has interesting parallels with the recently defined dual-role model of mTORC1 in physiological SC myelination (Beirowski et al., 2017; Figlia et al., 2017, 2018): (1) both in nerve development and regeneration, high mTORC1 activity is associated with a lower degree of SC differentiation; (2) in both cases, high mTORC1 activity inhibits differentiation to myelinating SCs, and thus a decline in mTORC1 activity is a prerequisite for the onset of both myelination and remyelination; and (3) in addition to inhibiting differentiation to myelinating SCs, high mTORC1 activity promotes processes specific to nerve development and regeneration, namely, radial sorting of axons in one case, myelin clearance and SC dedifferentiation in the other. Overall, a recurrent general theme emerges. By simultaneously inhibiting differentiation and promoting other processes, high mTORC1 activity ensures that the program of myelination (or remyelination) is halted until the other process has been completed, be it radial sorting or myelin clearance/demyelination. According to the dual-role model, the residual mTORC1 activity after onset of myelination supports myelin growth by promoting lipid (and likely protein) synthesis (Norrmén et al., 2014; Figlia et al., 2017). Although plausible, it remains experimentally uncertain whether the lower mTORC1 activity after onset of remyelination fulfills an analogous function in nerve regeneration. Indeed, the delayed demyelination and subsequent remyelination in RaptorKO nerves are also likely to contribute to a large extent to the radial hypomyelination that we detected at 60 dpc.

Ultimately, cell reprogramming entails changes in the expression or activity of transcription factors. The basic-leucine zipper transcription factor c-Jun has a major role in SC dedifferentiation and reprogramming to repair SCs (Arthur-Farraj et al., 2012, 2017; Jessen and Mirsky, 2016). However, which upstream signals are responsible for its rapid and strong upregulation in injured nerves is currently unclear. Although mTORC1 is considered to be required for efficient translation of virtually all mRNAs, not all mRNAs are equally dependent on mTORC1 for their translation (Thoreen et al., 2012). Among the mRNAs whose translation strongly depends on mTORC1, mRNAs encoding transcription factors have also been described (Ben-Sahra et al., 2016; Liu et al., 2017; Park et al., 2017). Here we provide evidence that mTORC1 signaling promotes c-Jun translation, in addition to a possible transcriptional control. Our data also suggest that the eIF4A subunit of eIF4F (Thoreen, 2013) might be involved in the translational control of c-Jun downstream of mTORC1. Thus, the rapid and strong upregulation of c-Jun upon nerve injury might encompass two synergistic processes: increased transcription through not yet identified mechanisms and increased translation via mTORC1 (Fig. 9). In line with this conclusion, we observed that: (1) c-Jun protein levels in explanted nerves increased also after blocking transcription; and (2) c-Jun protein levels upon injury increased significantly more than its mRNA levels. In addition to promoting SC dedifferentiation, c-Jun is also required for the emergence of repair SCs (Jessen and Mirsky, 2016). Consistent with this notion, the expression of genes specific to repair SCs was reduced in RaptorKO-injured nerves. Incidentally, we also showed that Runx2 is another marker for repair SCs whose function still needs to be explored. According to our experiments, nerve injury induces a general increase in protein synthesis, which can be prevented by mTORC1 inhibition. This indicates that also other mRNAs might depend on mTORC1 for their translation in dedifferentiating SCs. Further studies are warranted to determine the identities of these mRNAs and the function of the corresponding protein products in nerve injury. Consistent with the view that upon injury SCs undergo a process resembling epithelial-to-mesenchymal transition (Clements et al., 2017), an mTORC1-dependent increase in protein synthesis was also observed during classical epithelial-to-mesenchymal transition (Lamouille and Derynck, 2007; Lamouille et al., 2014).

The upstream signals activating mTORC1 in normal conditions remain to be exactly determined, but the SC growth factor neuregulin-1 (Nrg1) is likely involved (Heller et al., 2014; Figlia et al., 2017). However, whether changes in Nrg1 signaling alone can completely account for the variations of mTORC1 activity in developing SCs is unclear. Similarly, whether, and how, the same signals responsible for activating mTORC1 during development also contribute to mTORC1 reactivation upon injury is an unanswered question. In this context, the isoform Nrg1 Type I was shown to be strongly reexpressed in dedifferentiating SCs and to function as an autocrine signal (Stassart et al., 2013). Thus, an intriguing possibility is that induction of this isoform is responsible for the high mTORC1 activity observed in dedifferentiating SCs. Future studies are needed to confirm this hypothesis and to address whether and how mTORC1 activation accounts for the important functions of Nrg1 Type I in nerve regeneration.

Other signaling pathways have been studied in the context of SC plasticity (Harrisingh et al., 2004; Woodhoo et al., 2009; Napoli et al., 2012; Yang et al., 2012; Mogha et al., 2016; Clements et al., 2017), and some of them could directly or indirectly impact on mTORC1 activity. Erk1/2 phosphorylation increases very early after nerve injury and exuberant activation of this pathway caused SC dedifferentiation in uninjured nerves (Harrisingh et al., 2004; Napoli et al., 2012). As Erk1/2 are major upstream activators of the mTORC1 pathway (Sengupta et al., 2010), it is tempting to speculate that mTORC1 activation could, at least in part, account for the role of Mek-Erk1/2 in this context. Studies examining mTORC1 levels during Erk1/2-driven SC dedifferentiation, potentially followed by pharmacological inhibition of mTORC1, will be needed to address this possibility. Recently, TGFβ signaling has emerged as another major pathway for SC plasticity in injured nerves (Clements et al., 2017). Local release of TGFβ in injured nerves is required to reprogram SCs to a mesenchymal-like phenotype by regulating EphB2 signaling. Intriguingly, activation of mTORC1 downstream of TGFβ was reported to be involved in classical epithelial-to-mesenchymal transition (Lamouille and Derynck, 2007; Lamouille et al., 2014), suggesting it could similarly contribute to TGFβ-induced SC reprogramming.

Given the central role of c-Jun in the SC response to injury and the analogies between nerve injury and demyelinating neuropathies (Martini et al., 2013), modulation of c-Jun levels could have therapeutic applications in many pathological conditions of the PNS. c-Jun expression is increased in various peripheral neuropathies (Hutton et al., 2011), and its continuous expression in SCs appears to protect sensory axons from degeneration, as shown in animal models for a demyelinating neuropathy in which c-Jun was ablated (Hantke et al., 2014). However, moderate overexpression of c-Jun was also reported to delay remyelination after injury, and stronger overexpression substantially interfered also with developmental myelination (Fazal et al., 2017). Thus, a fine balance in c-Jun expression is required. By unveiling a link between mTORC1 and c-Jun expression, our present results suggest that mTORC1 inhibition with clinically approved drugs, such as rapamycin and rapamycin analogs (Benjamin et al., 2011), could be used to restore normal c-Jun levels in pathological conditions. In combination with the previously reported increase in Krox20 upon mTORC1 inhibition (Figlia et al., 2017), this effect may be of therapeutic relevance to restrict demyelination and favor remyelination, and may in part underlie the beneficial consequences of rapamycin administration in models of demyelinating neuropathies (Rangaraju et al., 2010; Nicks et al., 2014).
