# Supplementary figures and images for: mTORC1 Is Transiently Reactivated in Injured Nerves to Promote c-Jun Elevation and Schwann Cell Dedifferentiation
Source: J Neurosci. 2018 May 16;38(20):4811–28. doi: 10.1523/JNEUROSCI.3619-17.2018 (PMC5956991; doi:10.1523/JNEUROSCI.3619-17.2018)

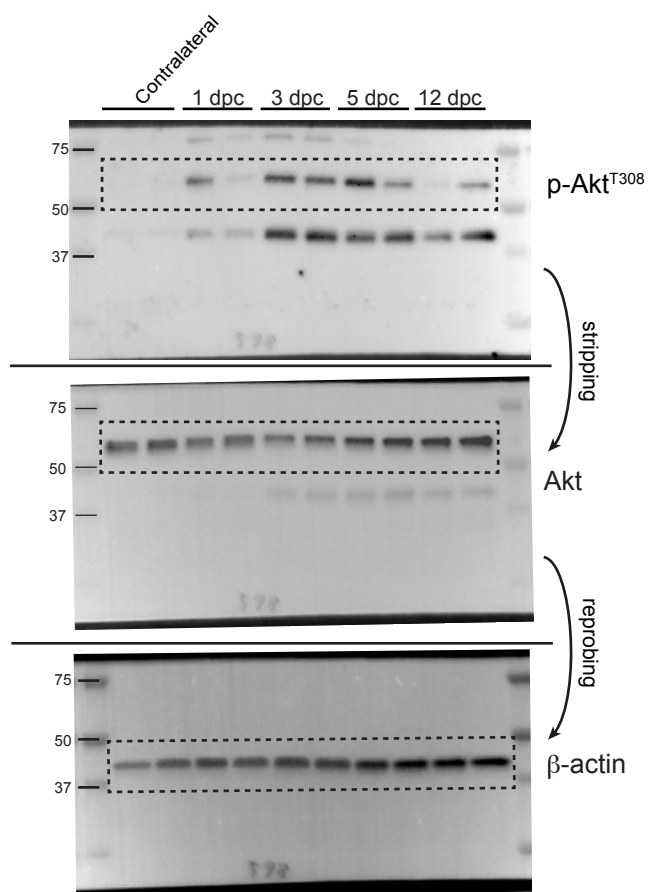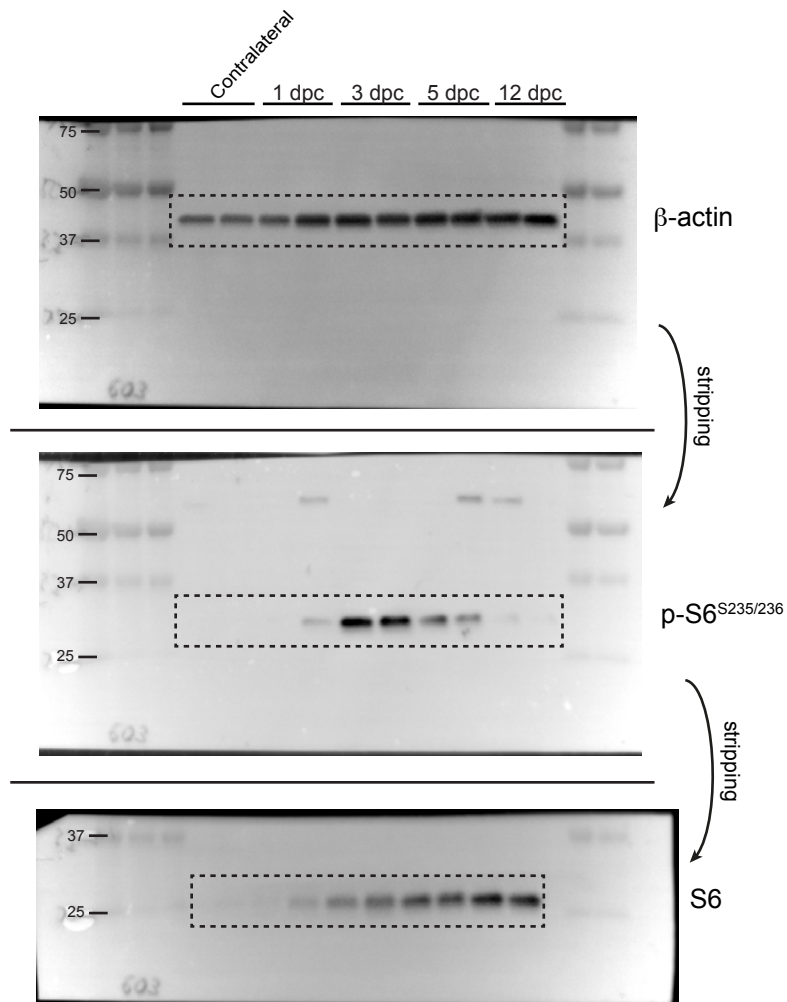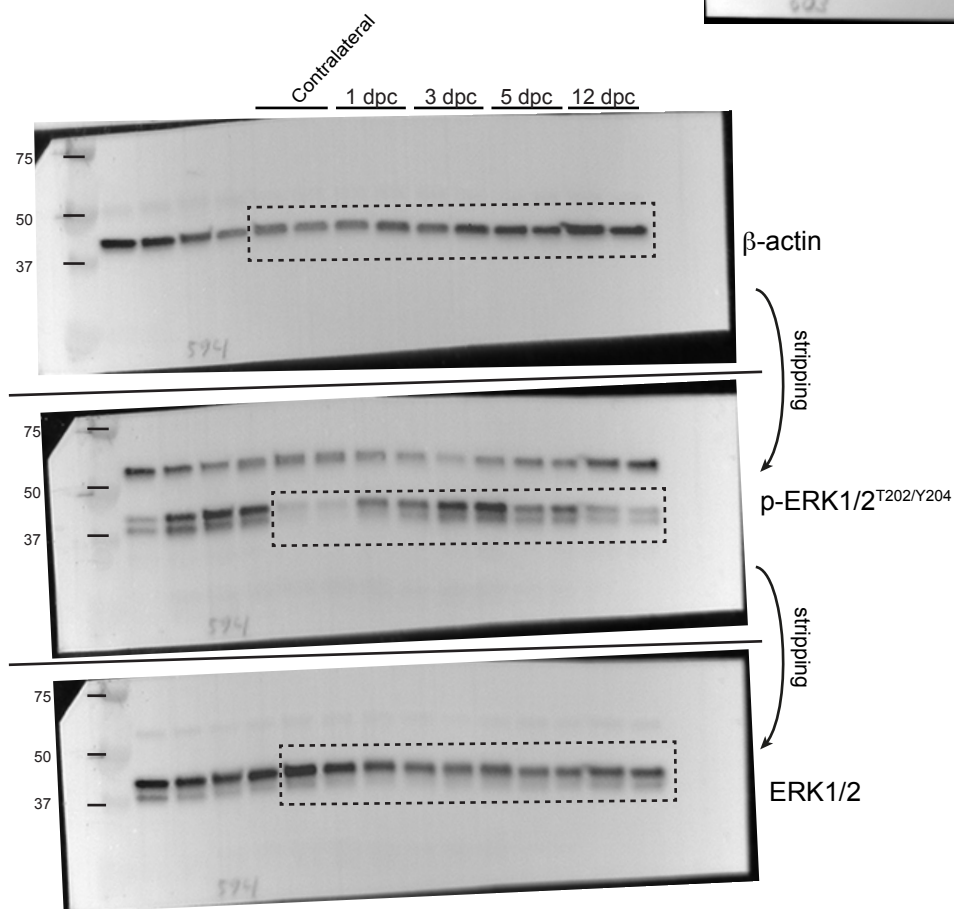

Figure 1-1

Supplement: Figure 1-1 [file zns999180787so1.pdf]

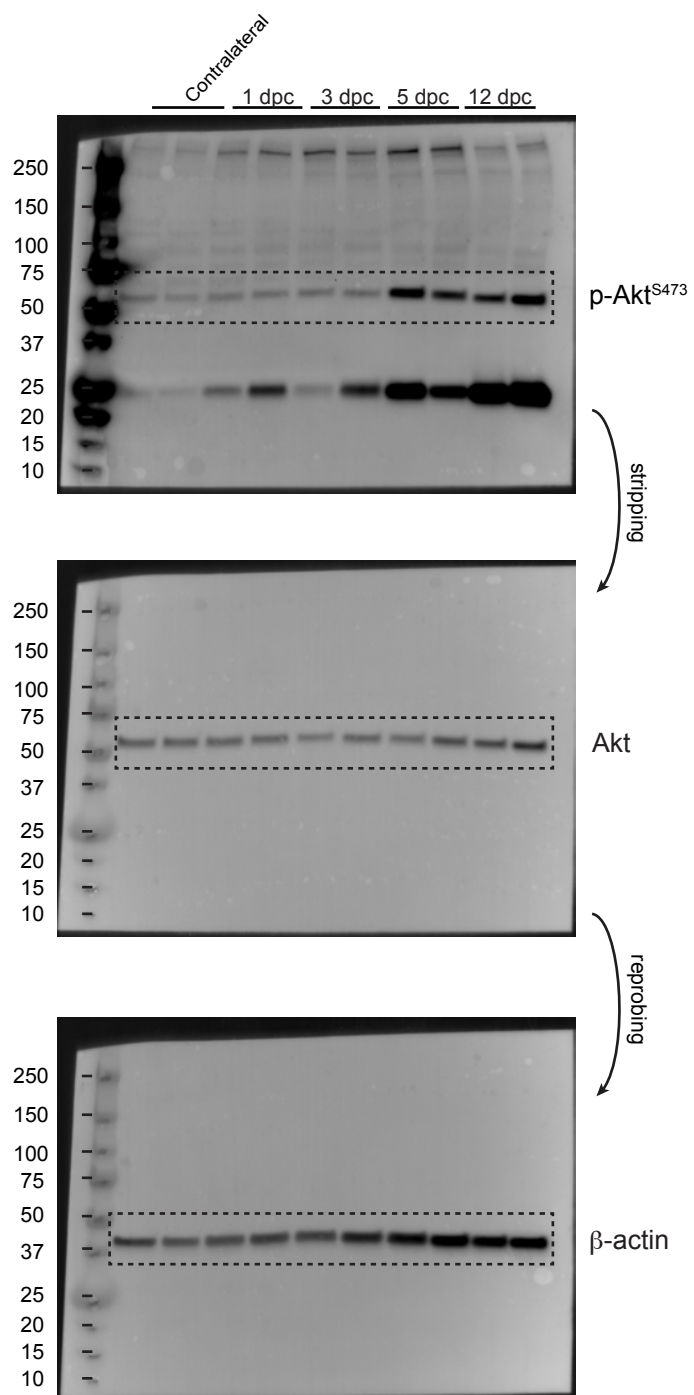

Figure 1-2

Supplement: Figure 1-2 [file zns999180787so2.pdf]

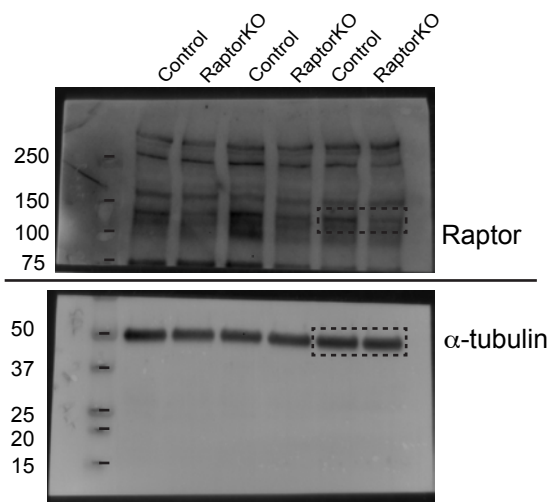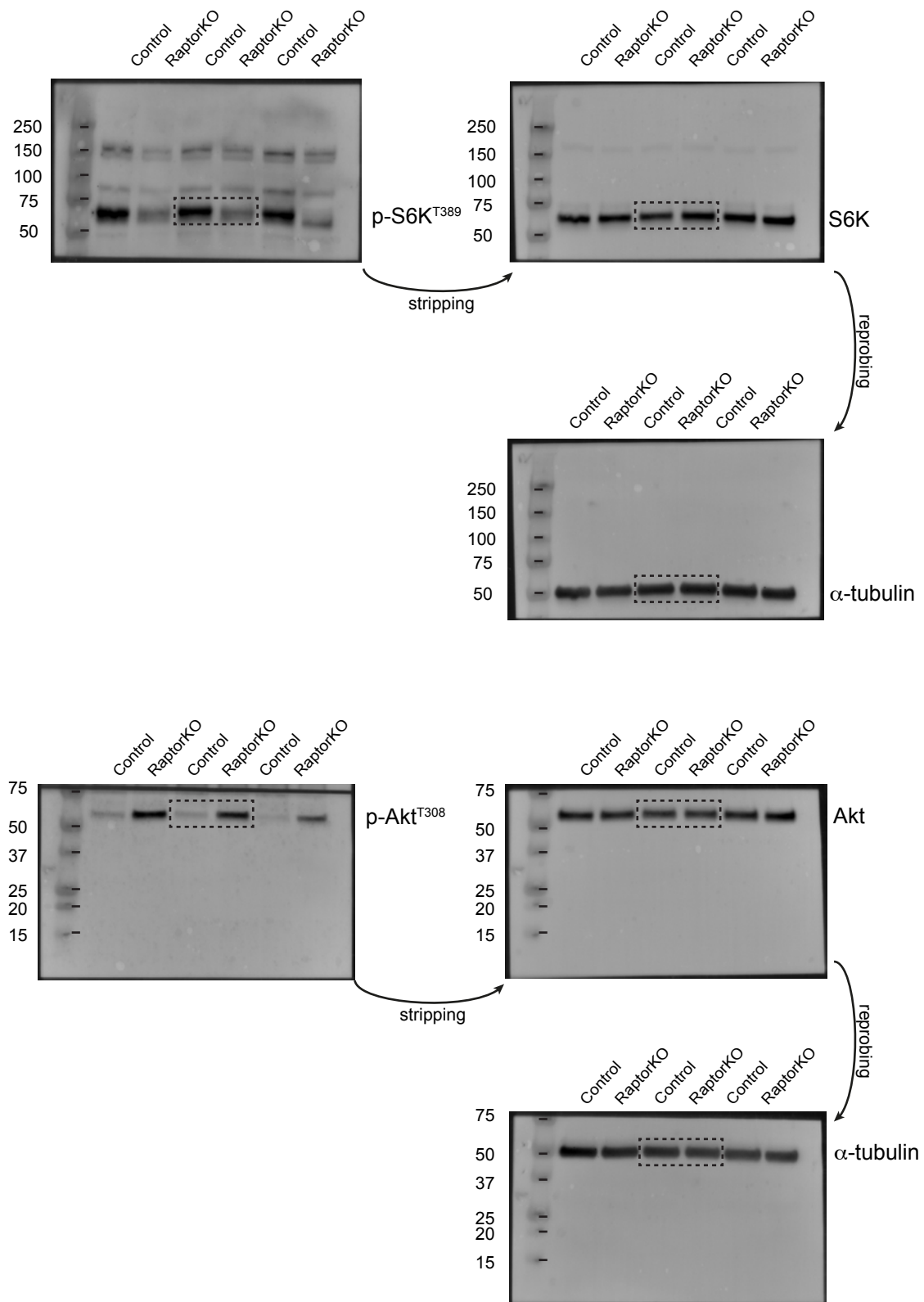

Figure 2-1

Supplement: Figure 2-1 [file zns999180787so3.pdf]

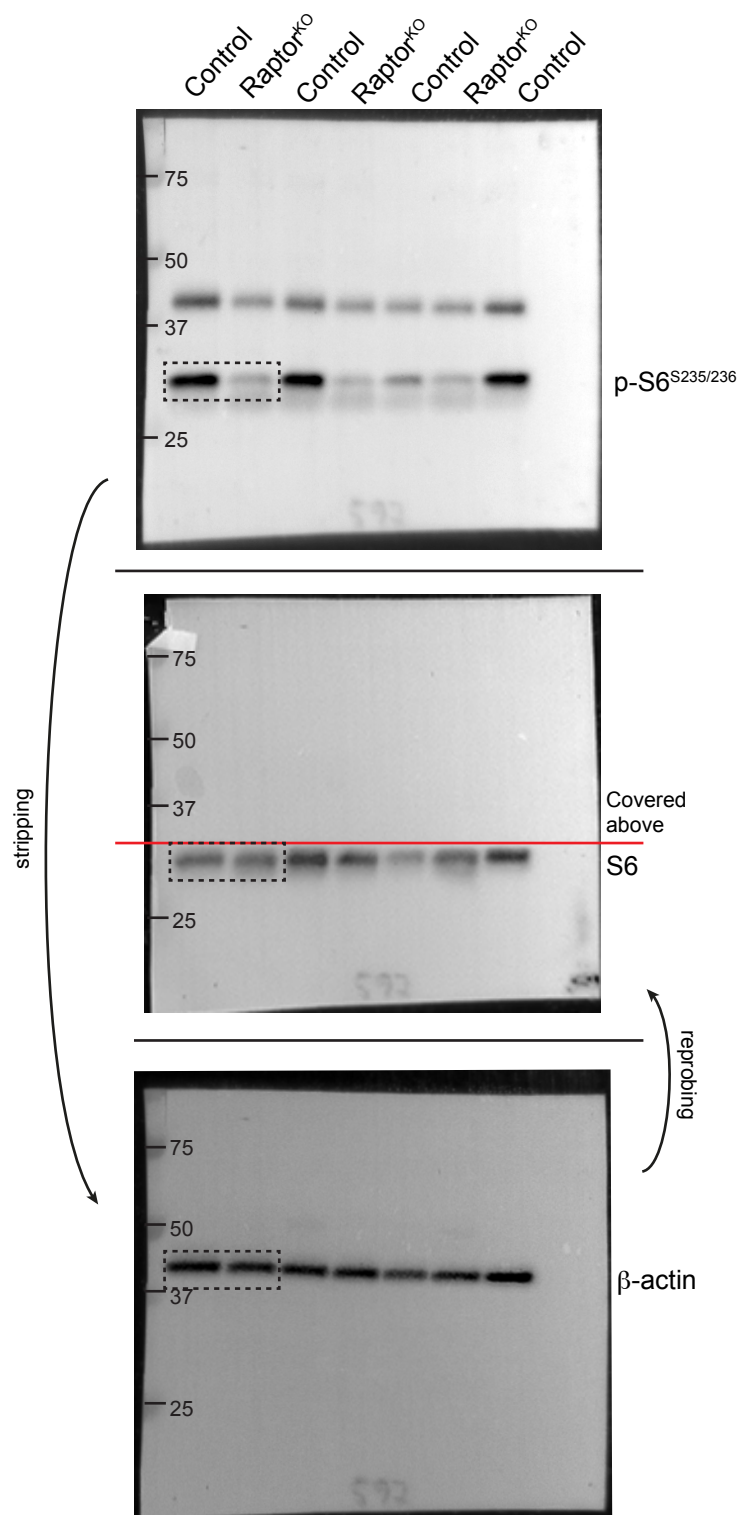

Figure 2-2

Supplement: Figure 2-2 [file zns999180787so4.pdf]

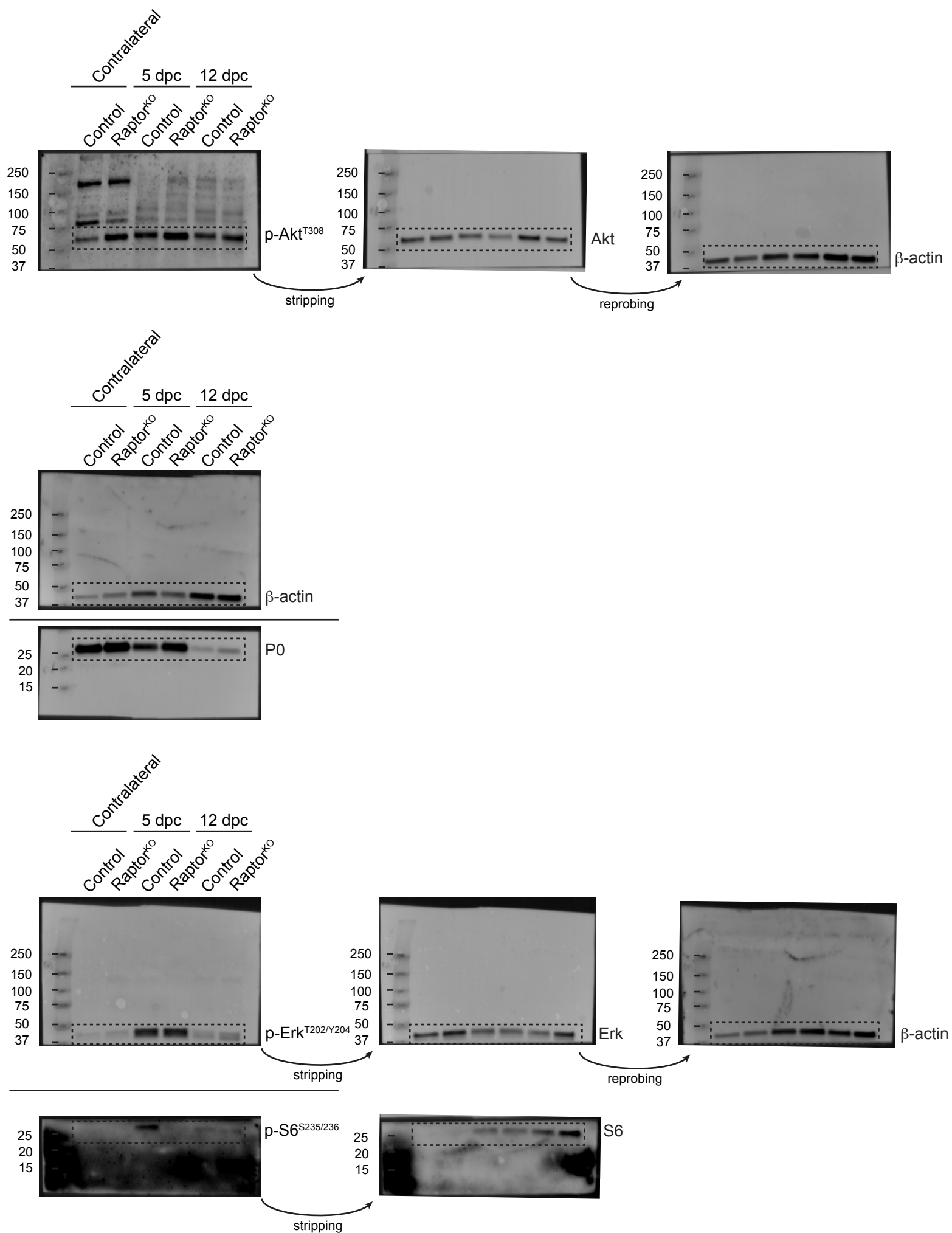

Figure 5-1

Supplement: Figure 5-1 [file zns999180787so5.pdf]

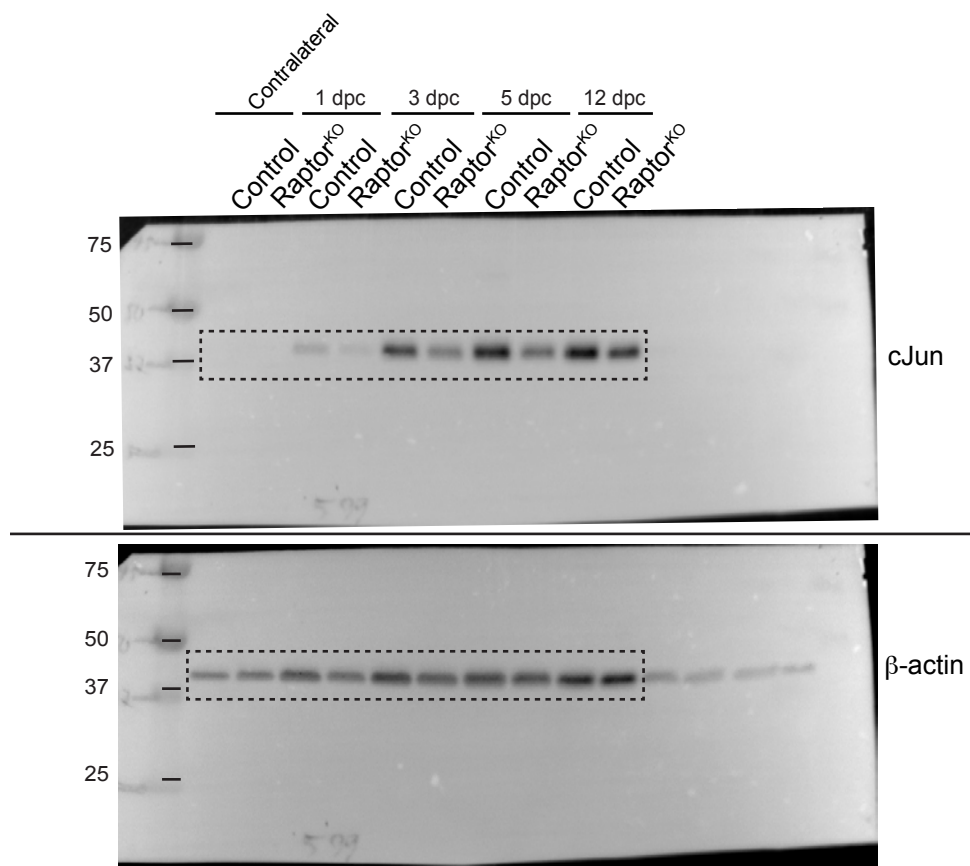

Figure 7-1

Supplement: Figure 7-1 [file zns999180787so7.pdf]

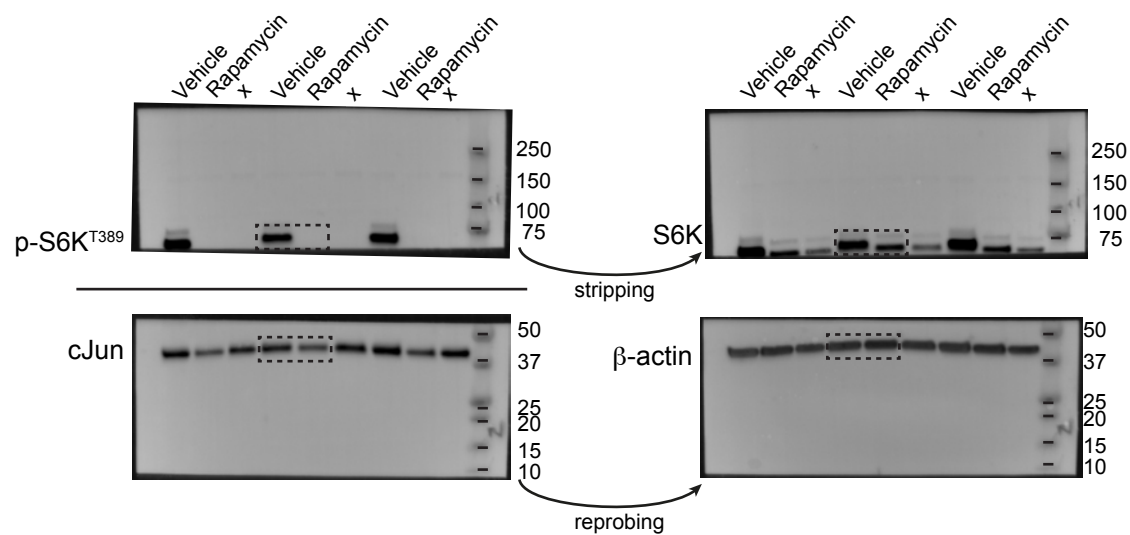

Figure 8-1

Supplement: Figure 8-1 [file zns999180787so8.pdf]

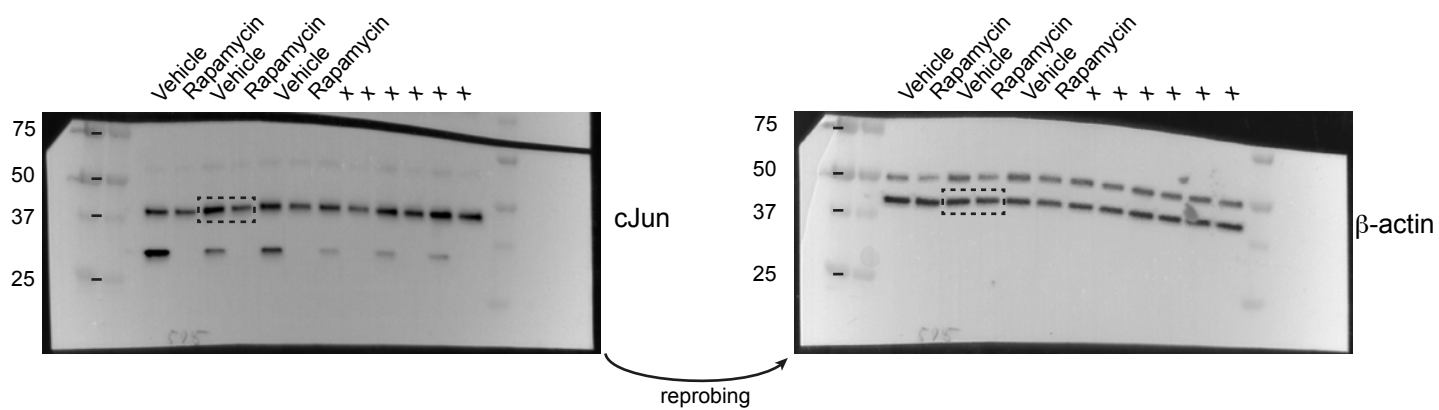

Figure 8-2

Supplement: Figure 8-2 [file zns999180787so9.pdf]

|           |   |   |   |   |   |   |   |   |   |
|-----------|---|---|---|---|---|---|---|---|---|
| Rapamycin | - | - | + | - | - | + | - | - | + |
| MG132     | - | + | + | - | + | + | - | + | + |

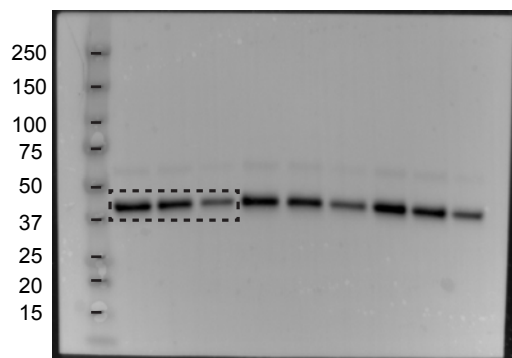

cJun

|           |   |   |   |   |   |   |   |   |   |
|-----------|---|---|---|---|---|---|---|---|---|
| Rapamycin | - | - | + | - | - | + | - | - | + |
| MG132     | - | + | + | - | + | + | - | + | + |

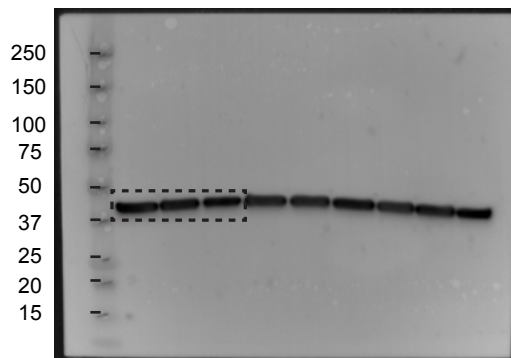

$\beta$ -actin

reprobing

Figure 8-3

Supplement: Figure 8-3 [file zns999180787so10.pdf]

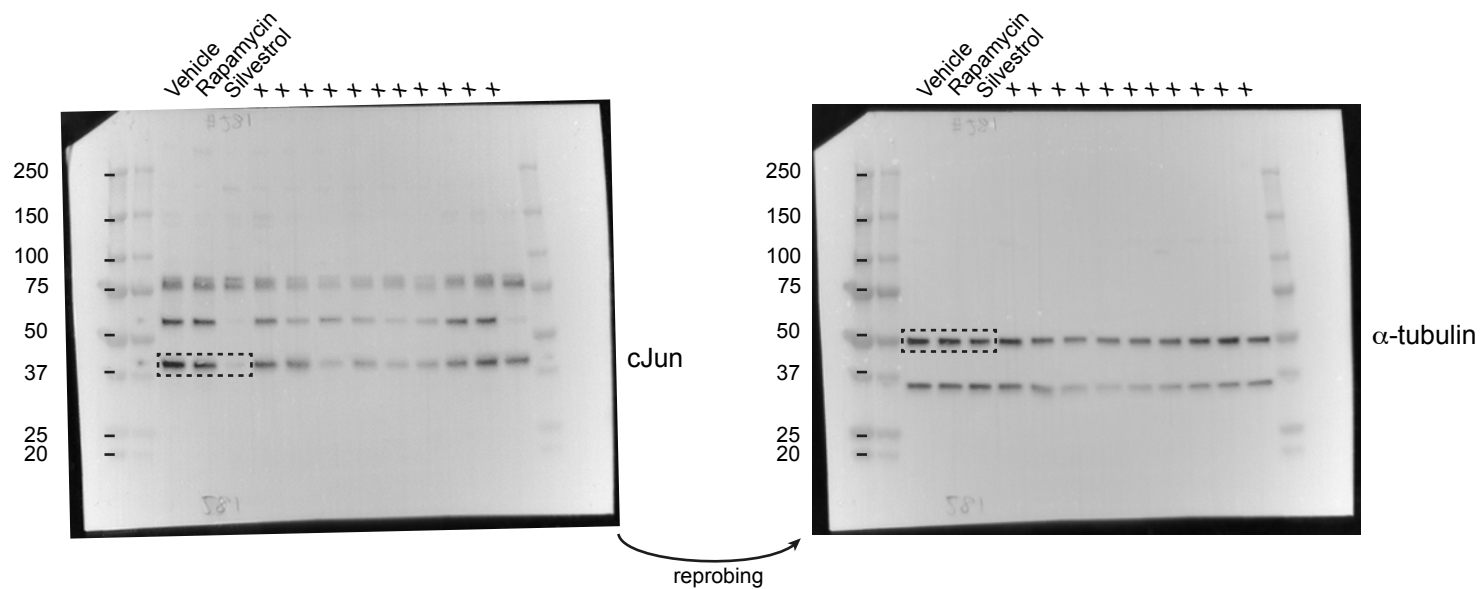

Figure 8-4

Supplement: Figure 8-4 [file zns999180787so11.pdf]

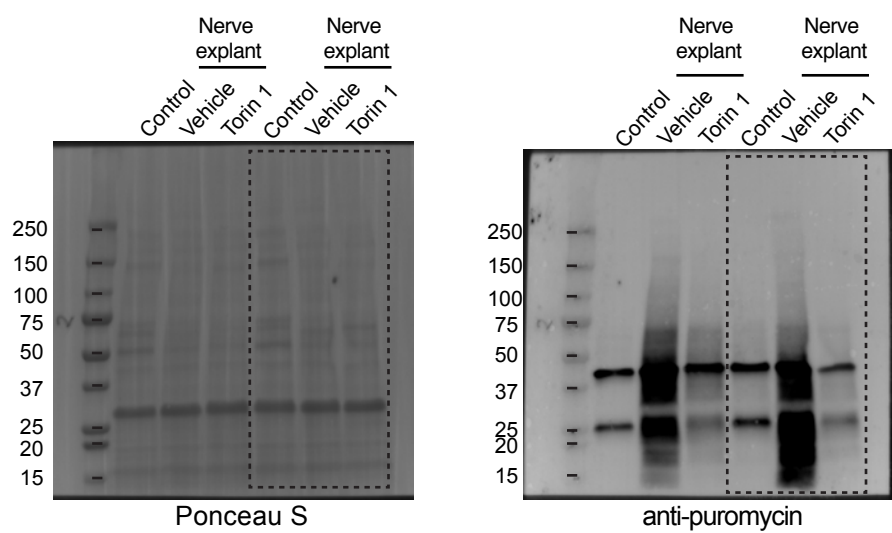

Figure 8-5

Supplement: Figure 8-5 [file zns999180787so12.pdf]

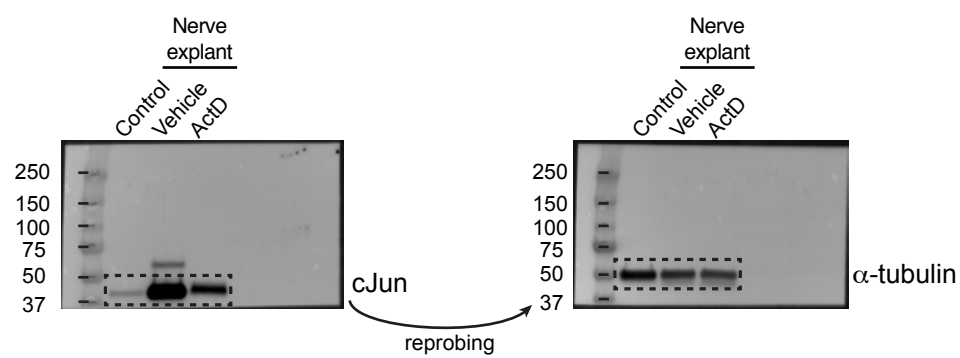

Figure 8-6

Supplement: Figure 8-6 [file zns999180787so13.pdf]
